# Predicting Left Ventricular Ejection Fraction Recovery After Percutaneous Coronary Intervention in Patients With Chronic Coronary Syndrome by Using Interpretable Machine Learning Models: Retrospective Study

**DOI:** 10.2196/77839

**Published:** 2025-12-29

**Authors:** Jiayi Ding, Guanqi Lyu, Masaharu Nakayama, Kotaro Nochioka, Jun Takahashi, Satoshi Yasuda, Tetsuya Matoba, Takahide Kohro, Naoyuki Akashi, Hideo Fujita, Yusuke Oba, Tomoyuki Kabutoya, Kazuomi Kario, Yasushi Imai, Arihiro Kiyosue, Yoshiko Mizuno, Takamasa Iwai, Yoshihiro Miyamoto, Masanobu Ishii, Kenichi Tsujita, Taishi Nakamura, Hisahiko Sato, Ryozo Nagai

**Affiliations:** 1 Department of Medical Informatics Graduate School of Medicine Tohoku University Sendai Japan; 2 Division of Cardiovascular Medicine Tohoku University Hospital Sendai Japan; 3 Department of Cardiovascular Medicine Graduate School of Medical Sciences Kyushu University Fukuoka Japan; 4 Department of Clinical Informatics School of Medicine Jichi Medical University Tochigi Japan; 5 Division of Cardiovascular Medicine Jichi Medical University Saitama Medical Center Saitama Japan; 6 Department of Medicine School of Medicine Jichi Medical University Tochigi Japan; 7 Department of Pharmacology Division of Clinical Pharmacology Jichi Medical University Tochigi Japan; 8 Department of Cardiovascular Medicine University of Tokyo Hospital Tokyo Japan; 9 Development Bank of Japan Tokyo Japan; 10 Department of Cardiovascular Medicine National Cerebral and Cardiovascular Center Suita Japan; 11 Open Innovation Center National Cerebral and Cardiovascular Center Suita Japan; 12 Department of Cardiovascular Medicine Graduate School of Medical Sciences Kumamoto University Kumamoto Japan; 13 Department of Medical Information Science Graduate School of Medical Sciences Kumamoto University Kumamoto Japan; 14 Precision Inc. Tokyo Japan; 15 School of Medicine Jichi Medical University Tochigi Japan

**Keywords:** chronic coronary syndrome, left ventricular ejection fraction, percutaneous coronary intervention, machine learning, prognostic modeling

## Abstract

**Background:**

Accurately predicting left ventricular ejection fraction (LVEF) recovery after percutaneous coronary intervention (PCI) in patients with chronic coronary syndrome (CCS) is crucial for clinical decision-making.

**Objective:**

This study aimed to develop and compare multiple machine learning (ML) models to predict LVEF recovery and identify key contributing features.

**Methods:**

We retrospectively analyzed 520 patients with CCS from the Clinical Deep Data Accumulation System database. Patients were categorized into 4 binary classification tasks based on baseline LVEF (≥50% or <50%) and degree of recovery: (1) good recovery, defined as an LVEF increase of >10% compared with ≤0%; and (2) normal recovery, defined as an LVEF increase of 0% to 10% compared with ≤0%. For each task, 3 feature selection strategies (all features, least absolute shrinkage and selection operator [LASSO] regression, and recursive feature elimination [RFE]) were combined with 4 ML algorithms (extreme gradient boosting [XGBoost], categorical boosting, light gradient boosting machine, and random forest), resulting in 48 models. Models were evaluated using 10-fold cross-validation and assessed by the area under the curve (AUC), decision curve analysis, and calibration plots.

**Results:**

The highest AUCs were achieved by RFE combined with XGBoost (AUC=0.93) for preserved LVEF with good recovery, LASSO combined with XGBoost (AUC=0.79) for preserved LVEF with normal recovery, LASSO combined with XGBoost (AUC=0.88) for reduced LVEF with good recovery, and RFE combined with XGBoost (AUC=0.84) for reduced LVEF with normal recovery. Shapley Additive Explanation analysis identified uric acid, platelets, hematocrit, brain natriuretic peptide, glycated hemoglobin, glucose, creatinine, baseline LVEF, left ventricular end-diastolic internal diameter, heart rate, R wave amplitude in V5, and R wave amplitude in V6 as important predictive factors of LVEF recovery.

**Conclusions:**

ML models incorporating feature selection strategies demonstrated strong predictive performance for LVEF recovery after PCI. These interpretable models may support clinical decision-making and can improve the management of patients with CCS after PCI.

## Introduction

With changing lifestyle habits and aging populations, coronary artery disease (CAD) has emerged as a leading global threat to health and mortality. It is projected that CAD will cause 7.8 million deaths worldwide by 2025 [1-4]. CAD is generally categorized into acute coronary syndrome (ACS) or chronic coronary syndrome (CCS) [5]. Among these, CCS is characterized by stable but progressive myocardial ischemia, presenting substantial challenges in long-term management and risk stratification [6].

Percutaneous coronary intervention (PCI), a mainstay treatment for CAD, aims to restore myocardial perfusion and is extensively used in patients with CCS [7]. However, despite successful revascularization, not all patients experience improved cardiac function, leading to poor long-term prognosis [8,9]. Recovery and remodeling of ventricular function critically influence PCI outcomes, and left ventricular ejection fraction (LVEF) is a key metric for assessing these changes [10-12]. Dynamic changes in LVEF are closely associated with myocardial recovery, heart failure incidence, hospital readmission, and cardiovascular mortality [13-15]. Therefore, identifying patients with varying degrees of LVEF recovery after PCI is essential for early risk stratification and personalized clinical management.

Traditionally, research on post-PCI recovery has focused primarily on patients with reduced LVEF, while those with preserved baseline LVEF have received less attention [16,17]. However, several studies have shown that a large proportion patient with preserved LVEF progress to reduced LVEF during follow-up, and this transition is usually associated with a worse clinical prognosis [14,18]. Thus, stratified modeling based on baseline LVEF is essential to capture the heterogeneity of post-PCI recovery and to enable early identification of potential high-risk patients.

In recent years, machine learning (ML) has been increasingly applied to PCI outcome prediction. However, most existing studies have focused on ACS populations and short-term events, such as major adverse cardiovascular events, mortality, or readmission [19-23]. By contrast, limited studies and datasets exist for the CCS population, in which post-PCI myocardial recovery plays a central role in long-term prognosis. Moreover, few studies have addressed the heterogeneity across baseline LVEF subgroups or incorporated interpretable ML approaches to elucidate the determinants of ventricular recovery. Therefore, this study aimed to develop and validate ML-based predictive frameworks to assess LVEF recovery within 6 to 12 months after PCI in patients with CCS with different baseline LVEF levels. Furthermore, we aimed to identify key factors influencing LVEF recovery to optimize post-PCI follow-up and treatment strategies, ultimately enhancing the long-term prognosis of patients with CCS.

## Methods

### Data Source

This study used the Clinical Deep Data Accumulation System (CLIDAS) database. Briefly, CLIDAS involves 6 university hospitals and 1 national center hospital in Japan, namely, Tohoku University, Jichi Medical University, Jichi Medical University Saitama Medical Center, University of Tokyo, National Cerebral and Cardiovascular Center Hospital, Kyushu University, and Kumamoto University [24,25]. The database includes data on patients’ backgrounds, blood test results, medication prescriptions, echocardiography data, electrocardiogram data, long-term prognosis, and information on cardiac catheterization [26].

### Ethical Considerations

This retrospective study was reviewed and approved by the Institutional Review Board of Jichi Medical University Hospital, Jichi Medical University, Tochigi, Japan (reference 23-158), and was conducted in accordance with the Declaration of Helsinki. The requirement for written informed consent was waived owing to the retrospective study design. All patient information was anonymized before analysis. JD and GL had full access to all the data in the study and take responsibility for its integrity and the data analysis.

### Study Population

We retrospectively analyzed patients with CCS who underwent PCI between April 2013 and December 2018 and were registered in the CLIDAS database. The inclusion criteria were as follows: (1) patients diagnosed with CCS and undergoing PCI for reasons other than ACS; (2) at least one echocardiogram showing LVEF within 30 days before PCI, that is, baseline LVEF (including day 30 before PCI and the day of PCI); (3) at least one echocardiogram showing LVEF during follow-up (180-365 days after PCI), that is, follow-up LVEF (including days 180 and 365 after PCI); and (4) available blood test data, demographics, electrocardiogram records, echocardiography examination records, cardiac catheterization insertion reports within 1 month before PCI (including day 30 before PCI and the day of PCI), and medication records within 10 days of PCI and before PCI. In total, 520 patients were included.

### Group Classification and Definition

The patients were grouped according to the change in their LVEF values (ΔLVEF; ΔLVEF=follow-up LVEF−baseline LVEF) to specifically characterize the improvement or deterioration of LVEF after PCI. For baseline and follow-up LVEF, only values closest to days 0 and 180, respectively, were considered. Good LVEF recovery was defined as ΔLVEF>10%, normal recovery as ΔLVEF>0% to ≤10%, and nonrecovery as ΔLVEF≤0%. Although the most recent European Society of Cardiology guidelines have introduced a new subtype of heart failure with a mild reduction in ejection fraction (ie, LVEF 40%-49%), this subtype is uncertain and is underrepresented in many clinical trials. In addition, owing to observer variability during echocardiographic procedures, this subtype is more difficult to differentiate and can lead to serious misclassifications.

Both American and Australian heart failure guidelines prefer to categorize heart failure with preserved ejection fraction and heart failure with reduced ejection fraction using a 50% LVEF threshold [27]. Therefore, in this study, the 50% baseline LVEF threshold was used to classify the patients into the preserved LVEF group (ie, LVEF≥50%) and the reduced LVEF group (ie, LVEF<50%). Subsequently, different degrees of LVEF recovery were combined with the 2 aforementioned groups, resulting in 4 distinct groups. These groups were used for model development and validation, with the aim of investigating the similarities and differences in factors influencing the varying degrees of LVEF recovery in these patients.

### Predictive Variables

A total of 131 predictor variables were analyzed. These included blood test data, demographic data, electrocardiogram recordings, echocardiogram recordings, cardiac catheterization reports within 30 days before PCI, and medication records within 10 days before PCI. Given that the same patient may have multiple measurements for each predictor variable, except for medication records, the one closest to the date of PCI was used. The number of medications was very large; thus, the medications were divided into categories to narrow them down, and the categories were converted into categorical variables using one-hot encoding. As the vascular locations of PCI implementation vary in cardiac catheterization reports, namely, the right coronary artery (RCA), left main trunk (LMT), left anterior descending artery (LAD), and left circumflex artery (LCX), 4 new binary variables were created to indicate the PCI implementation site: site_RCA, site_LMT, site_LAD, and site_LCX.

### Data Preprocessing

We first examined the distribution of missing data across the entire cohort and found no apparent systematic pattern. Therefore, data processing was conducted under an approximately missing at random assumption.

Variables with more than 30% missing data were removed. For variables with 10% to 30% data missingness, multivariate imputation by chained equations was applied, while variables with <10% data missingness were imputed using the median and mode value [28].

### Feature Selection

Three feature selection strategies were applied: all 131 variables (no selection), least absolute shrinkage and selection operator (LASSO) regression, and recursive feature elimination (RFE). LASSO selects features by penalizing the coefficients of less important variables, whereas RFE iteratively removes the least important features based on model performance. Both LASSO and RFE were implemented with embedded cross-validation to ensure the robustness and stability of feature selection across data partitions.

### Model Development and Evaluation

We constructed predictive models for each task using 4 ML algorithms: extreme gradient boosting (XGBoost), CatBoost, LightGBM, and random forest. Models were trained using 10-fold cross-validation to mitigate overfitting. Hyperparameters were optimized using the Optuna Bayesian optimization framework (Preferred Networks). Model performance was primarily evaluated by the receiver operating characteristic (ROC) and area under the curve (AUC), complemented by decision curve analysis (DCA) and calibration plots.

### Model Interpretability

To interpret and visualize model predictions, we used Shapley Additive Explanation (SHAP), which quantifies the contribution of each feature to the model’s predictions, facilitating clinical interpretability.

### Statistical Analysis

For baseline characteristics, categorical variables were expressed as percentages and compared between the groups using the chi-square test. The Kolmogorov-Smirnov test was used to assess the normality of the distribution of continuous variables. Normally distributed continuous variables were presented as the mean (SD) and compared using 2-tailed Student *t* test. Nonnormally distributed continuous variables were presented as the median (IQR) and compared using the Mann-Whitney *U* test. All statistical analyses and the model construction process were performed using Jupyter Notebook 6.4.8 (Project Jupyter) and Anaconda Navigator 2.3.2 (Anaconda Inc). The programming language used was Python 3.9.12. Statistical significance was set at *P*<.05, and it refers to the predefined threshold.

## Results

### Baseline Characteristics

We included 520 patients from the CLIDAS database. The average age of the total population was 71 (SD 10.49) years, and 401 (77.1%) patients were male. Diabetes mellitus and hypertension were present in 200 (38.5%) patients and 431 (82.9%) patients, respectively. According to the baseline LVEF, 418 (80.38%) patients belonged to the preserved LVEF group and 102 (19.61%) to the reduced LVEF group. The distribution of patients across baseline LVEF and recovery categories is presented in Table 1. The baseline characteristics of the preserved and reduced LVEF groups are summarized in Tables S1 and S2 in Multimedia Appendix 1, respectively. Owing to the large number of clinical variables, representative variables from each category are presented here. In the preserved LVEF group, hemoglobin, hematocrit (Hct), left ventricular end-systolic internal diameter, and baseline LVEF were substantially different between patients with and without functional recovery. In the reduced LVEF group, serum creatinine (Cre) and glucose (GLU) demonstrated sizable differences between recovery and nonrecovery patients.

**Table 1:** Group inclusion (N=520).

Group			Recovery, n (%)		Nonrecovery, n (%)
**Baseline LVEF^a^ ≥50%**					
	Preserved LVEF with good recovery^b^ (n=229)	43 (8.26)		186 (35.77)	
	Preserved LVEF with normal recovery^c^ (n=375)	189 (36.35)		186 (35.77)	
**Baseline LVEF<50%**					
	Reduced LVEF with good recovery (n=68)	44 (8.46)		24 (4.61)	
	Reduced LVEF with normal recovery (n=58)	34 (6.54)		24 (4.61)	

^a^LVEF: left ventricular ejection fraction.

^b^Good recovery: an LVEF increase of >10% compared with ≤0%.

^c^Normal recovery: an LVEF increase of 0% to 10% compared with ≤0%.

### Predictive Performance of the Models

A total of 48 predictive models were developed by combining 3 feature selection strategies (all features, LASSO, and RFE) with 4 ML algorithms (XGBoost, CatBoost, LightGBM, and random forest) across 4 prediction tasks. Model performance evaluated using the AUC score is summarized in Table 2. Additional details, including task-specific feature selection, ROC curves, calibration, and DCA plots of all models, as well as other performance metrics for the best-performing models are presented in Figures S1-S3 and Table S3 in Multimedia Appendix 1.

**Table 2:** The area under the curve score of each model.

		XGBoost^a^	CatBoost^b^	LightGBM^c^	RF^d^
**pG^e^**					
	All features	0.90	0.89	0.87	0.86
	LASSO^f^	0.89	0.90	0.87	0.87
	RFE^g^	0.93	0.92	0.90	0.88
**pN^h^**					
	All features	0.76	0.75	0.75	0.70
	LASSO	0.79	0.77	0.75	0.72
	RFE	0.77	0.74	0.75	0.72
**rG^i^**					
	All features	0.83	0.81	0.76	0.60
	LASSO	0.88	0.81	0.80	0.82
	RFE	0.85	0.82	0.83	0.74
**rN^j^**					
	All features	0.81	0.83	0.77	0.66
	LASSO	0.82	0.81	0.78	0.72
	RFE	0.84	0.83	0.77	0.68

^a^XGBoost: extreme gradient boosting.

^b^CatBoost: categorical boosting.

^c^LightGBM: light gradient boosting machine.

^d^RF: random forest.

^e^pG: preserved left ventricular ejection fraction with good recovery.

^f^LASSO: least absolute shrinkage and selection operator.

^g^RFE: recursive feature elimination.

^h^pN: preserved left ventricular ejection fraction with normal recovery.

^i^rG: reduced left ventricular ejection fraction with good recovery.

^j^rN: reduced left ventricular ejection fraction with normal recovery.

The best-performing models for each task were as follows:

- Preserved LVEF with good recovery: RFE+XGBoost (AUC=0.93)
- Preserved LVEF with normal recovery: LASSO+XGBoost (AUC=0.79)
- Reduced LVEF with good recovery: LASSO+XGBoost (AUC=0.88)
- Reduced LVEF with normal recovery: RFE+XGBoost (AUC=0.84)

**Figure 1:**
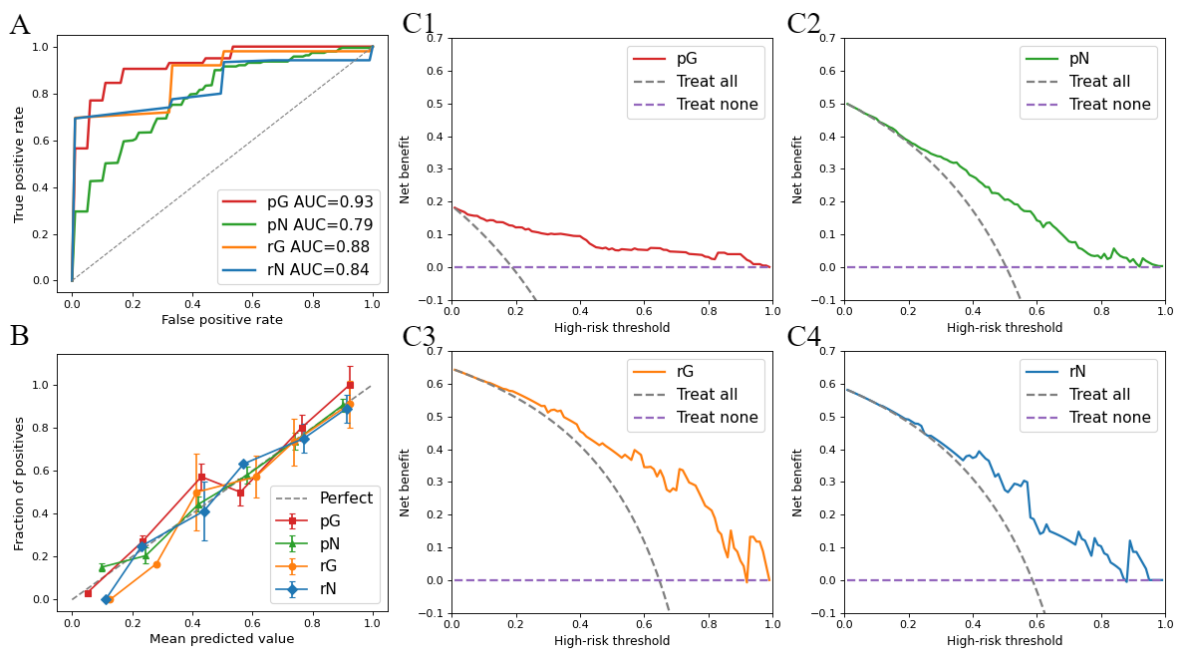
Model performance of each task: (A) receiver operating characteristic curves for optimal models in each task, (B) calibration curve of the 4 optimal models, and (C1-C4) clinical decision curves of the optimal models for each task. AUC: area under the curve; pG: preserved left ventricular ejection fraction with good recovery task; pN: preserved left ventricular ejection fraction with normal recovery task; rG: reduced left ventricular ejection fraction with good recovery task; rN: reduced left ventricular ejection fraction with normal recovery task.

Figure 1 shows the performance of the optimal models for each task. ROC curves demonstrated strong discriminative power across tasks, especially in predicting patient with good recovery tasks. Calibration plots showed the alignment of predicted probabilities with observations for different risk levels, providing a visual assessment of how well each model’s predicted risk fits with actual event rates. DCA plots revealed substantial clinical net benefit of the optimal models compared to treat-all and treat-none strategies across a wide range of threshold probabilities, supporting their potential clinical applicability.

Subgroup analyses stratified by sex, age, and comorbidities demonstrated consistent model performance across clinically relevant patient groups (Table S4 in Multimedia Appendix 1).

### SHAP Summary Plot

**Figure 2:**
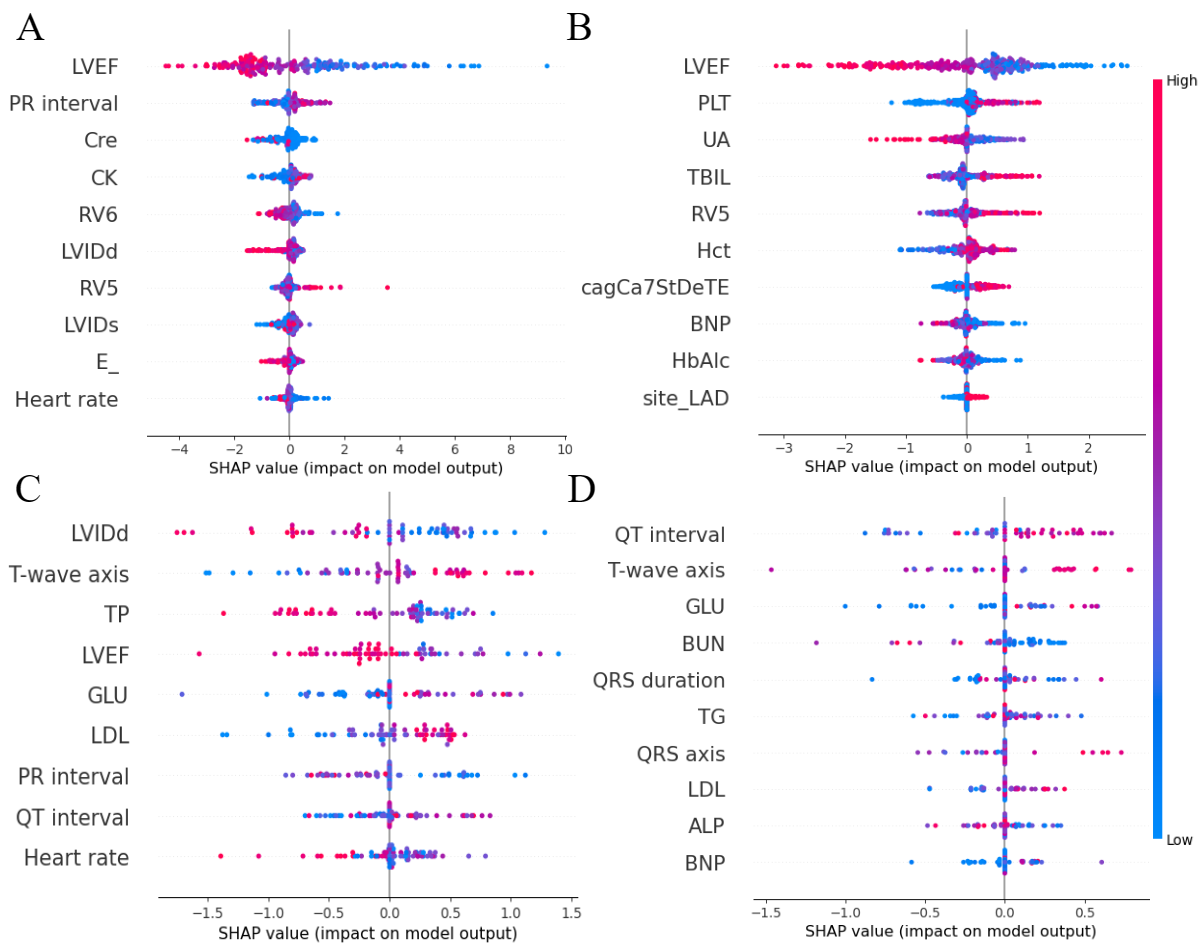
Top important features identified by Shapley Additive Explanations (SHAP): (A) important features in the preserved left ventricular ejection fraction with good recovery task, (B) important features in the preserved left ventricular ejection fraction with normal recovery task, (C) important features in the reduced left ventricular ejection fraction with good recovery task, and (D) important features in the reduced left ventricular ejection fraction with normal recovery task. ALP: alkaline phosphatase; BNP: brain natriuretic peptide; BUN: blood urea nitrogen; cagCa7StDeTE: stenosis degree of coronary artery segment 7; CK: creatine kinase; Cre: creatinine; E_: mitral E wave velocity; GLU: glucose; HbA_1c_: glycated hemoglobin; Hct: hematocrit; LAD: left anterior descending artery; LDL: low-density lipoprotein; LVEF: left ventricular ejection fraction; LVIDd: left ventricular end-diastolic internal diameter; LVIDs: left ventricular end-systolic internal diameter; PLT: platelet; RV5: R wave amplitude in V5; RV6: R wave amplitude in V6; TBIL: total bilirubin; TG: triglycerides; TP: total protein; UA: uric acid.

SHAP is a graphical method for interpreting ML models based on the concept of Shapley values in game theory. Each line in the SHAP summary plot represents a predictor variable, with variables that have a greater impact on the model output being closer to the top. The SHAP summary plots for the 4 optimal models are shown in Figure 2. Overall, some cardiac function–related parameters were identified as important. These included baseline LVEF; left ventricular end-diastolic internal diameter (LVIDd); R wave amplitude in V5 (RV5) or R wave amplitude in V6 (RV6); heart rate; and some laboratory parameters, including uric acid (UA), platelets (PLTs), Hct, brain natriuretic peptide (BNP), glycated hemoglobin (HbA_1c_), GLU, and Cre.

## Discussion

### Principal Findings

In this study, our models showed satisfactory performance across all groups after hyperparameter optimization and 10-fold cross-validation. The results indicated the strong discriminatory ability of ML models in predicting the recovery status of LVEF within 6 months to 1 year after PCI in patients with CCS. The application of feature selection strategies, such as RFE and LASSO, effectively improved model performance. Furthermore, UA, PLTs, Hct, BNP, HbA_1c_, GLU, Cre, baseline LVEF, LVIDd, heart rate, RV5, and RV6 were identified as important predictors of post-PCI LVEF recovery in patients with CCS with different baseline LVEF levels. These findings can help clinicians better understand the factors influencing LVEF recovery, thereby supporting timely adjustments in treatment and follow-up strategies to improve patient outcomes.

Compared with previous studies, our work presents several methodological and clinical advancements. From a clinical perspective, our study moves beyond the traditional focus on adverse end points. Previous ML-based PCI studies have primarily focused on ACS populations and short-term event outcomes such as major adverse cardiovascular events or mortality [19-23], which capture only terminal prognostic states. In contrast, post-PCI LVEF recovery reflects the intermediate phase of cardiac repair, serving as a mechanistically meaningful and clinically actionable indicator of long-term prognosis. We focused on the 6- to 12-month post-PCI period, which captures the critical window of myocardial remodeling while minimizing interference from immediate or distant postoperative events. As most improvement or decline in LVEF occurs within this period [12,17], early prediction of recovery potential during this phase enables timely adjustment of medical therapy and secondary prevention strategies, providing a more proactive framework for improving patient outcomes [29]. Furthermore, our study addresses the real-world heterogeneity of CCS by uniquely including patients across both reduced and preserved LVEF subgroups [30,31], thereby covering a broader clinical spectrum that was often overlooked in previous research. Methodologically, we used a more rigorous and transparent framework. Earlier post-PCI studies mainly relied on single statistical models or isolated ML algorithms, often neglecting subgroup variability and the robustness of feature selection [16,32,33]. In contrast, our framework systematically compared different feature selection methods (RFE and LASSO) and integrated SHAP analysis to enhance interpretability. This combination improved model robustness clarified the contribution of individual predictors, and strengthened clinical transparency and trust.

Interestingly, models generally perform better with larger, more balanced datasets [34]. However, in our study, the best-performing task was predicting good recovery in preserved LVEF, rather than the task with the largest and most balanced sample size. The results remained consistent after accounting for class imbalance through category weighting sensitivity analysis (Table S5 in Multimedia Appendix 1). In addition, the good recovery task was superior to the normal recovery task across different baseline LVEF groups, indicating that patients with substantial LVEF improvement after PCI may have more defined potential patterns and clinical characteristics. This phenomenon is consistent with previous studies, suggesting that clear patient characteristics typically yield higher prediction accuracy even with smaller datasets [35,36]. Identifying these unique clinical trajectories and patterns can accurately stratify patients, leading to targeted interventions and improved clinical management.

Currently, clinical practice and research in post-PCI management focus on patients with reduced LVEF due to their substantial myocardial dysfunction, while largely ignoring patients with preserved LVEF. However, a *normal* LVEF does not necessarily reflect normal myocardial function. Previous studies have shown that patients with preserved LVEF may exhibit abnormal myocardial strain, elevated ventricular filling pressures, and an increased risk of adverse outcomes [37-39]. Yoshihisa et al [18] reported that approximately 30% of patients with preserved LVEF had a reduction in LVEF to ≤50% within 6 months, which was associated with poorer clinical outcomes. Similarly, another longitudinal study showed that a significant proportion of patients with preserved LVEF converted to reduced LVEF during a 3-year follow-up [40]. These results highlight the importance of promptly and accurately predicting LVEF trends in patients with preserved LVEF after PCI. Our model provides a practical tool for the early detection of potentially high-risk patients with preserved LVEF, which can improve their long-term prognosis through close monitoring of key factors and timely intervention.

In this study, SHAP analysis identified several key predictors of LVEF recovery, including laboratory parameters (UA, PLT, Hct, BNP, HbA_1c_, GLU, Cre), cardiac function–related variables (baseline LVEF, LVIDd, and heart rate), and electrocardiogram parameters such as RV5 and RV6. Among laboratory and metabolic indicators, higher UA levels were associated with predicted nonrecovery, consistent with the role of hyperuricemia in promoting oxidative stress, endothelial dysfunction, and inflammation that drive adverse ventricular remodeling [41,42]. This aligns with the findings of Akashi et al [25], who identified hyperuricemia as a risk factor for heart failure after PCI in patients with CCS. Low Hct impairs oxygen delivery, and Wang et al [43] found that Hct levels were significantly lower in patients with CAD than in healthy controls. Interestingly, higher PLT counts were positively associated with recovery probability. This finding may reflect the reparative function of PLTs beyond thrombosis, as they release bioactive mediators, including growth factors and cytokines, that promote myocardial repair, angiogenesis, and inflammatory resolution [44]. Beyond these hematologic and metabolic markers, cardiovascular and systemic biomarkers also aid in risk stratification. Elevated BNP reflects neurohormonal activation and ventricular wall stress and has been linked to adverse outcomes even in patients with preserved LVEF after revascularization [45,46]. Increased HbA_1c_ indicates chronic metabolic stress and impaired myocardial recovery potential, consistent with studies showing that poor glycemic control limits functional improvement after PCI in both diabetic and nondiabetic populations [47,48]. Elevated Cre, as a marker of renal dysfunction [49], was also correlated with impaired cardiac functional recovery [50]. GLU also emerged as an important predictor in some subgroups. As the myocardium relies predominantly on GLU oxidation for energy, moderate elevations in GLU may reflect adequate metabolic substrate availability, consistent with the positive association observed in our model [51].

Regarding cardiac structural and functional parameters, an enlarged LVIDd was negatively associated with recovery, reflecting impaired ventricular compliance and chronic remodeling, as also shown by Chen et al [52]. Higher resting heart rate predicted poorer recovery, plausibly because increased sympathetic activation elevates myocardial oxygen demand and exacerbates ischemic stress, consistent with the findings of Lei et al [2].

For electrocardiographic parameters, the SHAP analysis indicated that higher RV6 amplitudes and lower RV5 amplitudes were predictive of nonrecovery. These voltage patterns are typical of left ventricular hypertrophy and electrical remodeling, both of which reduce compliance and delay reverse remodeling after revascularization [42]. Repolarization features, such as longer QT intervals and greater T-wave axis deviations, were positively associated with recovery in the reduced LVEF group, possibly reflecting adaptive electrical remodeling during functional improvement.

Importantly, lesion-specific variables, such as stenosis degree of coronary artery segment 7 (cagCa7StDeTE) and site_LAD, were positively associated with predicted recovery in the preserved LVEF with normal recovery group. This positive association does not imply a beneficial effect of severe stenosis but rather reflects the efficacy of complete and timely revascularization in critical myocardial territory. Proximal LAD lesions compromise a large myocardial territory and are critical determinants of left ventricular function; therefore, successful PCI in these segments can lead to substantial recovery [45]. Patients in our cohort with severe LAD stenosis who underwent PCI demonstrated superior LVEF recovery. This suggests that anatomically targeted revascularization meaningfully contributes to reverse remodeling, even in the preserved function group. The inclusion of these lesion-level features underscores our dataset’s unique strength. It shows that the model captures not only systemic and biochemical predictors but also procedural and anatomical factors. This provides an additional layer of interpretability that has been rarely addressed in prior studies.

### Limitations

First, the relatively small sample size and the inclusion of only patients with complete echocardiographic follow-up data may have introduced selection bias, although this was necessary to ensure outcome accuracy. Second, although the dataset was derived from a multicenter real-world cohort in Japan, external validation was not conducted due to the specificity of included variables, limiting generalizability. Third, as a real-world cohort, unavoidable data missingness existed. Removing variables with a high proportion of missing data may have affected model performance. Furthermore, if some variables were missing not at random, this could affect feature selection or model performance.

Future work should expand the cohort and include external datasets for validation, while prospectively collecting standardized data to allow a more rigorous evaluation of missingness mechanisms and model generalizability. Moreover, developing dynamic models to predict continuous changes in LVEF and integrating additional modalities, such as imaging and biomarkers, may further enhance predictive accuracy and clinical utility.

### Conclusions

Our ML models, which integrated feature selection strategies, demonstrated strong predictive performance for LVEF recovery after PCI. SHAP analysis identified UA, PLT, Hct, BNP, HbA_1c_, GLU, Cre, baseline LVEF, LVIDd, heart rate, RV5, and RV6 as key factors of post-PCI LVEF recovery.

## Appendix

Multimedia Appendix 1 Additional results of various analyses.

## Abbreviations

ΔLVEF: change in left ventricular ejection fraction value
ACS: acute coronary syndrome
AUC: area under the curve
BNP: brain natriuretic peptide
CAD: coronary artery disease
CatBoost: categorical boosting
CCS: chronic coronary syndrome
CLIDAS: Clinical Deep Data Accumulation System
Cre: creatinine
DCA: decision curve analysis
GLU: glucose
HbA_1c_: glycated hemoglobin
Hct: hematocrit
LAD: left anterior descending artery
LASSO: least absolute shrinkage and selection operator
LightGBM: light gradient boosting machine
LMT: left main trunk
LVEF: left ventricular ejection fraction
LVIDd: left ventricular end-diastolic internal diameter
ML: machine learning
PCI: percutaneous coronary intervention
PLT: platelet
RCA: right coronary artery
RFE: recursive feature elimination
ROC: receiver operating characteristic
RV5: R wave amplitude in V5
RV6: R wave amplitude in V6
SHAP: Shapley Additive Explanation
UA: uric acid
XGBoost: extreme gradient boosting
