# Supplementary material for: Predicting Left Ventricular Ejection Fraction Recovery After Percutaneous Coronary Intervention in Patients With Chronic Coronary Syndrome by Using Interpretable Machine Learning Models: Retrospective Study
Source: JMIR Med Inform. 2025 Dec 29;13:e77839. doi: 10.2196/77839 (PMC12796882; doi:10.2196/77839)
Supplement: Multimedia Appendix 1 [file medinform_v13i1e77839_app1.docx]

**Supplementary materials**

[Table S1. Baseline characteristics of patients with preserved LVEF. 1](#_Toc216180642)

[Table S2. Baseline characteristics of patients with reduced LVEF. 4](#_Toc216180643)

[Table S3 Performance of Optimal Models 6](#_Toc216180644)

[Table S4 Subgroups Analysis 7](#_Toc216180645)

[Table S5 Class-weighting Sensitive Analysis 9](#_Toc216180646)

[Figure S1 Plots of each task using all feature 10](#_Toc216180647)

[Figure S2 Plots of each task using features selected by RFE 11](#_Toc216180648)

[Figure S3 Plots of each task using features selected by LASSO. 12](#_Toc216180649)

[Figure S4 Features selected by RFE 13](#_Toc216180650)

[Figure S5 Features selected by LASSO 14](#_Toc216180651)

#

# Table S1. Baseline characteristics of patients with preserved LVEF.

| Variables | Total  (*N*=418) | Recovery group  (*N*=232) | Non-recovery group  (*N*=186) | *P* value |
| --- | --- | --- | --- | --- |
|  |  |  |  |  |
| **Clinicodemographic** |  |  |  |  |
| Age (years) | 71 (64-77) | 70 (64-76) | 71 (65-79) | .076 |
| Males (%) | 76.1 | 75.0 | 77.4 | .565 |
| Stroke (%) | 10.3 | 8.2 | 12.9 | .116 |
| Diabetes mellitus (%) | 37.3 | 35.8 | 39.3 | .379 |
| Atrial fibrillation (%) | 3.1 | 3.0 | 3.2 | .904 |
| Hypertension (%) | 83.3 | 81.0 | 86.0 | .176 |
| Dyslipidemia (%) | 82.1 | 82.8 | 81.2 | .677 |
| Hemodialysis (%) | 5.5 | 5.6 | 5.4 | .920 |
| BMI (kg/m^2^) | 23.9 (22.1-26.6) | 24.1 (22.2-26.6) | 23.7 (22.0-26.4) | .529 |
| **Laboratory** |  |  |  |  |
| ALB (g/dL) | 4.0 (3.7-4.3) | 4.1 (3.8-4.3) | 4.0 (3.6-4.3) | .064 |
| AST (U/L) | 20 (16-27) | 21 (16-28) | 19 (16-26) | .074 |
| ALT (U/L) | 17 (12-24) | 17 (13-25) | 16 (11-24) | .081 |
| ALP (U/L) | 219 (180-265) | 218 (180-260) | 220 (180-272) | .596 |
| BUN (mg/dL) | 17 (13- 21) | 17 (14-20) | 17 (13-23) | .591 |
| Cre (mg/dL) | 0.9 (0.8-1.1) | 0.9 (0.8-1.1) | 0.9 (0.8-1.2) | .141 |
| UA (mg/dL) | 5.7 (4.8-6.5) | 5.6 (4.7-6.3) | 5.7 (4.9-6.7) | .055 |
| CK (U/L) | 90.5 (61.0-131.0) | 94.0 (63.0-135.0) | 88.5 (59.0-120.8) | .356 |
| GLU (mg/dL) | 119 (100-146) | 119 (101-147) | 117 (99-145) | .487 |
| HbA1c (%) | 6.2 (5.8-6.9) | 6.1 (5.7-6.9) | 6.2 (5.8-6.9) | .117 |
| BNP (pg/mL) | 75 (30-208) | 77 (30-219) | 74 (27-192) | .980 |
| CRP (mg/dL) | 0.12 (0.04-0.32) | 0.13 (0.04-0.30) | 0.12 (0.05-0.33) | .497 |
| WBC (${10}^{3}$/μL) | 6.0 (5.1-7.7) | 6.0 (5.1-7.5) | 6.0 (5.0-7.8) | .858 |
| RBC (${10}^{6}$/μL) | 414.0 (369.5-456.0) | 404.0 (350.5-451.5) | 408.0 (360.3-452.0) | .633 |
| Hb (g/dL) | 12.9 (11.5-14.1) | 13.1 (11.9-14.3) | 12.6 (11.2-14.0) | .015 |
| Hct (%) | 39.1 (34.9-42.6) | 39.5 (35.9-42.8) | 38.1 (33.6-42.0) | .023 |
| MCH (pg) | 31.0 (30.0-32.2) | 31.1 (30.1-32.2) | 30.9 (29.8-32.3) | .146 |
| MCHC (g/dL) | 33.0 (32.5-33.6) | 33.1 (32.5-33.7) | 33.0 (32.5-33.6) | .665 |
| PLT (10^3^/μL) | 21.1 (16.8-146.0) | 21.7 (17.2-147.3) | 20.7 (15.6-142.5) | .222 |
| **Echocardiography** |  |  |  |  |
| IVST (mm) | 10.2 (9.0-11.5) | 10.1 (9.0-12.0) | 10.2 (9.0-11.4) | .814 |
| LVPWT (mm) | 10.1 (9.0-11.3) | 10.0 (9.0-11.3) | 10.4 (9.0-11.5) | .243 |
| LVIDd (mm) | 47.0 (43.0-51.0) | 47.9 (44.0-51.0) | 46.3 (42.0-51.1) | .232 |
| LVIDs (mm) | 30.0 (26.0-33.6) | 30.9 (27.2-34.4) | 28.5 (24.9-32.9) | <.001 |
| LAD (mm) | 39.2 (35.6-42.0) | 39.2 (35.0-42.0) | 39.2 (36.0-42.0) | .558 |
| LVEF (%) | 64.0 (58.0-69.0) | 61.6 (56.0-66.0) | 67.8 (61.4-74.0) | <.001 |
| E/A ratio | 0.80 (0.68-1.10) | 0.80 (0.69-1.08) | 0.80 (0.67-1.10) | .892 |
| **Electrocardiogram** |  |  |  |  |
| Heart rate (BPM) | 63.0 (57.0-71.0) | 63.0 (57.0-70.0) | 63.0 (57.0-72.0) | .323 |
| PR interval (ms) | 172.0 (158.0-188.0) | 172.0 (158.0-186.0) | 172.50 (158.0-189.8) | .475 |
| QRS duration (ms) | 99.0 (90.0-109.0) | 100.0 (90.0-109.0) | 98.0 (88.0-108.8) | .544 |
| QT interval (ms) | 414.0 (387.3-434.0) | 413.5 (388.0-434.0) | 414.0 (384.50-434.0) | .899 |
| RV5 (mV) | 1.73 (1.37-2.23) | 1.84 (1.41-2.30) | 1.67 (1.30-2.21) | .075 |
| RV5 + SV1 (mV) | 2.65 (2.08-3.31) | 2.67 (2.03-3.41) | 2.62 (2.13-3.25) | .658 |
| RV6 (mV) | 1.29 (1.10-1.41) | 1.28 (1.10 -1.41) | 1.30 (1.08-1.43) | .539 |
| RV6 + SV1 (mV) | 2.10 (1.43-3.17) | 2.08 (1.42-3.17) | 2.12 (1.45-3.16) | .992 |

Abbreviations: LVEF, left ventricular ejection fraction; BMI, body mass index; ALB, albumin; AST, aspartate aminotransferase; ALT, alanine aminotransferase; ALP, alkaline phosphatase; BUN, blood urea nitrogen; Cre, creatinine; UA, uric acid; CK, creatine kinase; GLU, glucose; HbA1c, glycosylated hemoglobin A1c; BNP, B-type natriuretic peptide; CRP, C-reactive protein; WBC, white blood cell; RBC, red blood cell; Hb, hemoglobin; Hct, hematocrit; MCH, mean corpuscular hemoglobin; MCHC, mean corpuscular hemoglobin concentration; PLT, platelet; IVST, interventricular septal thickness; LVPWT, left ventricular posterior wall thickness; LVIDd, left ventricular end-diastolic internal diameter; LVIDs: left ventricular end-systolic internal diameter; LAD, left atrial diameter; BPM, beats per minute; RV5, R wave amplitude in V5; SV1, S wave in V1; RV6, R wave amplitude in V6

# Table S2. Baseline characteristics of patients with reduced LVEF.

| Variables | Total  (N=102) | Recovery group  (N=78) | Non-recovery group  (N=24) | *P* value |
| --- | --- | --- | --- | --- |
|  |  |  |  |  |
| **Clinicodemographic** |  |  |  |  |
| Age (years) | 71 (65-78) | 70.50 (63 -79) | 69.00 (66-72) | .460 |
| Males (%) | 81.4 | 79.5 | 87.5 | .561 |
| Stroke (%) | 13.7 | 12.8 | 16.7 | .889 |
| Diabetes mellitus (%) | 42.2 | 42.3 | 41.7 | .990 |
| Atrial fibrillation (%) | 8.8 | 4.7 | 8.8 | .529 |
| Hypertension (%) | 81.4 | 83.0 | 88.2 | .636 |
| Dyslipidemia (%) | 80.4 | 80.8 | 79.2 | .962 |
| Hemodialysis (%) | 9.8 | 11.5 | 4.2 | .503 |
| BMI (kg/m^2^) | 23.0 (21.3-25.9) | 23.1 (21.1-26.1) | 22.8 (21.6-25.7) | .912 |
| **Laboratory** |  |  |  |  |
| ALB (g/dL) | 4.0 (3.5-4.2) | 4.0 (3.5-4.2) | 3.95 (3.6-4.2) | .725 |
| AST (U/L) | 21.0 (16.0-28.0) | 21.0 (16.3-28.0) | 19.0 (15.0-28.8) | .441 |
| ALT (U/L) | 17.0 (11.0-27.0) | 17.5 (10.3-27.0) | 17.0 (12.5-27.8) | .807 |
| ALP (U/L) | 235.5 (190.3-289.3) | 225.5 (184.8-286.8) | 255.0 (230.3-303.0) | .101 |
| BUN (mg/dL) | 19.0 (15.0-25.8) | 19.0 (14.3-23.0) | 20.0 (16.0-30.0) | .199 |
| Cre (mg/dL) | 1.0 (0.8-1.3) | 0.9 (0.8-1.2) | 1.1 (0.9-1.8) | .045 |
| UA (mg/dL) | 5.9 (4.7-7.2) | 5.9 (4.7-7.2) | 6.0 (4.7-7.1) | .859 |
| CK (U/L) | 73.0 (44.3-110.5) | 72.0 (42.3-112.8) | 74.5 (45.8-102.3) | .943 |
| GLU (mg/dL) | 115.5 (102.3-137.8) | 120.0 (103.3-143.3) | 105.5 (100.3-119.0) | .038 |
| HbA1c (%) | 6.2 (5.8-6.9) | 6.2 (5.8-6.8) | 6.2 (5.9-6.9) | .997 |
| BNP (pg/mL) | 266.5 (122.4-502.0) | 277.15 (113.0-488.3) | 249.0 (146.1-555.4) | .878 |
| CRP (mg/dL) | 0.2 (0.1-0.4) | 0.19 (0.1-0.4) | 0.17 (0.1-0.4) | .312 |
| WBC (${10}^{3}$/μL) | 6.6 (5.3-8.4) | 6.6 (5.4-8.5) | 6.39 (5.3-7.9) | .912 |
| RBC (${10}^{6}$/μL) | 416.0 (367.0- 451.3) | 420.0 (363.5-450.0) | 412.0 (370.3-449.9) | .759 |
| Hb (g/dL) | 12.8 (11.8-14.0) | 12.8 (11.8-14.1) | 12.75 (12.0-14.0) | .975 |
| Hct (%) | 38.9 (35.9-42.8) | 38.75 (35.9-43.0) | 39.15 (36.5-42.0) | .981 |
| MCH (pg) | 31.0 (29.5-31.9) | 31.0 (29.4-32.3) | 30.85 (30.0-31.5) | .679 |
| MCHC (g/dL) | 33.0 (32.3-33.5) | 32.9 (32.3-33.5) | 32.95 (31.8-33.4) | .622 |
| PLT (10^3^/μL) | 26.2 (17.9-191.0) | 27.5 (17.9-194.3) | 24.65 (18.0-160.3) | .984 |
| **Echocardiography** |  |  |  |  |
| IVST (mm) | 10.0 (9.0-11.0) | 10.0 (9.0-11.3) | 10.0 (9.0-10.2) | .609 |
| LVPWT (mm) | 10.0 (9.0-11.0) | 10.0 (9.0-11.0) | 10.1 (10.0-11.1) | .074 |
| LVIDd (mm) | 54.0 (48.8-61.0) | 53.9 (48.8-59.8) | 56.0 (50.3-63.0) | .168 |
| LVIDs (mm) | 42.8 (37.0-48.3) | 42.3 (36.2-48.8) | 44.5 (39.8-48.0) | .449 |
| LAD (mm) | 39.9 (39.0-45.8) | 39.7 (38.3-45.8) | 40.7 (39.3-44.8) | .528 |
| LVEF (%) | 40.0 (31.8-46.0) | 39.9 (31.2-46.0) | 44.0 (38.4-46.2) | .298 |
| E/A ratio | 1.1 (0.7-1.3) | 1.1 (0.8-1.3) | 1.2 (0.7-1.2) | .837 |
| **Electrocardiogram** |  |  |  |  |
| Heart rate (BPM) | 69.0 (63.0-77.8) | 69.0 (63.0-76.0) | 70.5 (62.8-84.5) | .330 |
| PR interval (ms) | 175.0 (164.0-193.5) | 174.0 (159.5-194.0) | 176.5 (172.8-187.0) | .348 |
| QRS duration (ms) | 101.5 (93.3-114.5) | 101.5 (95.0-112.3) | 103.5 (89.8-115.3) | .719 |
| QT interval (ms) | 418.0 (390.3-441.0) | 421.5 (396.0-441.5) | 398.5 (380.8-436.0) | .129 |
| RV5 (mV) | 1.25 (0.7-2.0) | 1.3 (0.7-2.0) | 1.0 (0.6-2.0) | .461 |
| RV5 + SV1 (mV) | 2.5 (1.8-3.5) | 2.5 (1.9-3.4) | 2.2 (1.4-3.5) | .708 |
| RV6 (mV) | 1.2 (0.7-1.3) | 1.2 (0.9-1.3) | 1.2 (0.5-1.5) | .834 |
| RV6 + SV1 (mV) | 2.2 (1.8-2.4) | 2.2 (1.8-2.4) | 2.2 (1.8-3.0) | .997 |

# Table S3 Performance of Optimal Models

| Task | AUC | Recall | Specificity | Precision | Confusion Matrix |
| --- | --- | --- | --- | --- | --- |
| pG | 0.93 | 0.70 | 0.97 | 0.83 | $\binom{180,6}{13,30}$ |
| pN | 0.79 | 0.74 | 0.70 | 0.71 | $\binom{130,56}{50,139}$ |
| rG | 0.88 | 0.98 | 0.63 | 0.83 | $\binom{15,9}{1,43}$ |
| rN | 0.84 | 0.91 | 0.67 | 0.79 | $\binom{16,8}{3,31}$ |

For each best-performing model, recall, specificity, precision and confusion matrix were calculated based on the 10-fold cross-validation results. Overall, the models maintained a reasonable balance between sensitivity (0.70–0.98) and specificity (0.63–0.97), reflecting reliable identification of both recovered and non-recovered patients. Slightly lower specificity in the rG and rN tasks likely reflects the smaller sample sizes and greater heterogeneity of reduced-LVEF groups, which tend to have overlapping clinical features.

# Table S4 Subgroups Analysis

| Task | Subgroup | Number | AUC | ΔAUC |
| --- | --- | --- | --- | --- |
| pG | Male | 176 | 0.88 | -0.05 |
|  | Female | 53 | 0.99 | +0.06 |
|  | Age>=65 | 171 | 0.9 | -0.03 |
|  | Age<65 | 58 | 0.93 | 0 |
|  | Hypertension | 197 | 0.89 | -0.04 |
|  | No-Hypertension | 32 | 1.0 | +0.07 |
|  | Diabetes | 87 | 0.86 | -0.07 |
|  | No-Diabetes | 142 | 0.93 | 0 |
| pN | Male | 286 | 0.77 | -0.02 |
|  | Female | 89 | 0.84 | +0.05 |
|  | Age>=65 | 278 | 0.78 | -0.01 |
|  | Age<65 | 97 | 0.78 | -0.01 |
|  | Hypertension | 311 | 0.79 | 0 |
|  | No-Hypertension | 64 | 0.74 | -0.05 |
|  | Diabetes | 144 | 0.78 | -0.01 |
|  | No-Diabetes | 231 | 0.78 | -0.01 |
| rG | Male | 12 | 0.82 | -0.06 |
|  | Female | 56 | 0.89 | +0.01 |
|  | Age>=65 | 54 | 0.83 | -0.05 |
|  | Age<65 | 14 | 0.84 | -0.04 |
|  | Hypertension | 57 | 0.87 | -0.01 |
|  | No-Hypertension | 11 | 0.92 | +0.04 |
|  | Diabetes | 32 | 0.92 | +0.04 |
|  | No-Diabetes | 36 | 0.87 | -0.01 |
| rN | Male | 48 | 0.81 | -0.03 |
|  | Female | 10 | 0.86 | +0.02 |
|  | Age>=65 | 41 | 0.80 | -0.04 |
|  | Age<65 | 17 | 0.88 | +0.04 |
|  | Hypertension | 47 | 0.84 | 0 |
|  | No-Hypertension | 11 | 0.73 | -0.11 |
|  | Diabetes | 21 | 0.85 | +0.01 |
|  | No-Diabetes | 37 | 0.80 | -0.04 |

Each prediction task (pG, pN, rG, rN) was stratified by sex (male vs female), age (≥65 vs <65 years), hypertension (yes/no), and diabetes (yes/no). For each subgroup, the area under the ROC curve (AUC) was calculated using the same trained model as in the main analysis. To quantify subgroup variation, the ΔAUC was defined as subgroup AUC minus overall AUC.

Overall, the models demonstrated good robustness with most ΔAUC values within ±0.05, indicating stable predictive capability across different patient subgroups. Specifically, female patients showed slightly higher AUCs which may reflect smaller ventricular volumes and more homogeneous post-PCI recovery patterns, leading to clearer separation between good and poor outcomes. Similarly, younger patients tended to have marginally higher AUCs, possibly due to a lower comorbidity burden and more consistent physiological responses to PCI. In contrast, the non- hypertension subgroups occasionally exhibited extreme AUCs (e.g., pG AUC = 1.00, N=32; rN AUC = 0.73, N=11.); however, these strata contained very small sample sizes, and such fluctuations are most likely attributable to sampling variability rather than genuine biological differences.

Taken together, these findings indicate that the models maintain robust and fair discrimination across demographic and comorbidity-defined patient groups.

# Table S5 Class-weighting Sensitive Analysis

|  | AUC | Weighted AUC | ΔAUC |
| --- | --- | --- | --- |
| pG | 0.93 | 0.91 | -0.02 |
| pN | 0.79 | 0.77 | -0.02 |
| rG | 0.88 | 0.88 | 0 |
| rN | 0.84 | 0.81 | -0.03 |

To further evaluate the potential influence of imbalance, we additionally trained XGBoost models using class weighting (scale_pos_weight) and compared the results with those of the unweighted models. The performance difference was minimal across the four best-performing models (ΔAUC ≤ 0.03 across tasks), suggesting that class imbalance did not materially affect model performance.

#

# Figure S1 Plots of each task using all feature


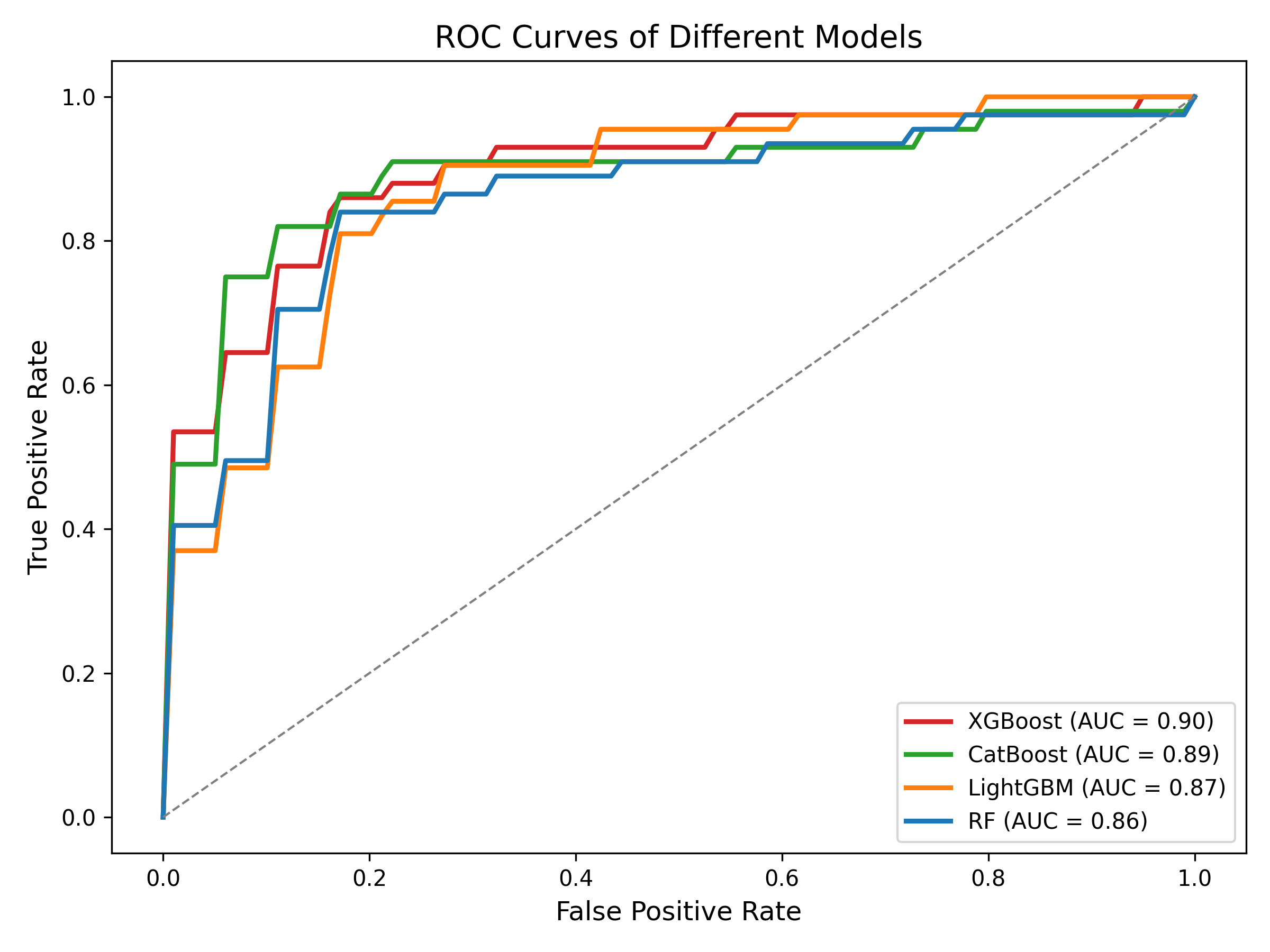

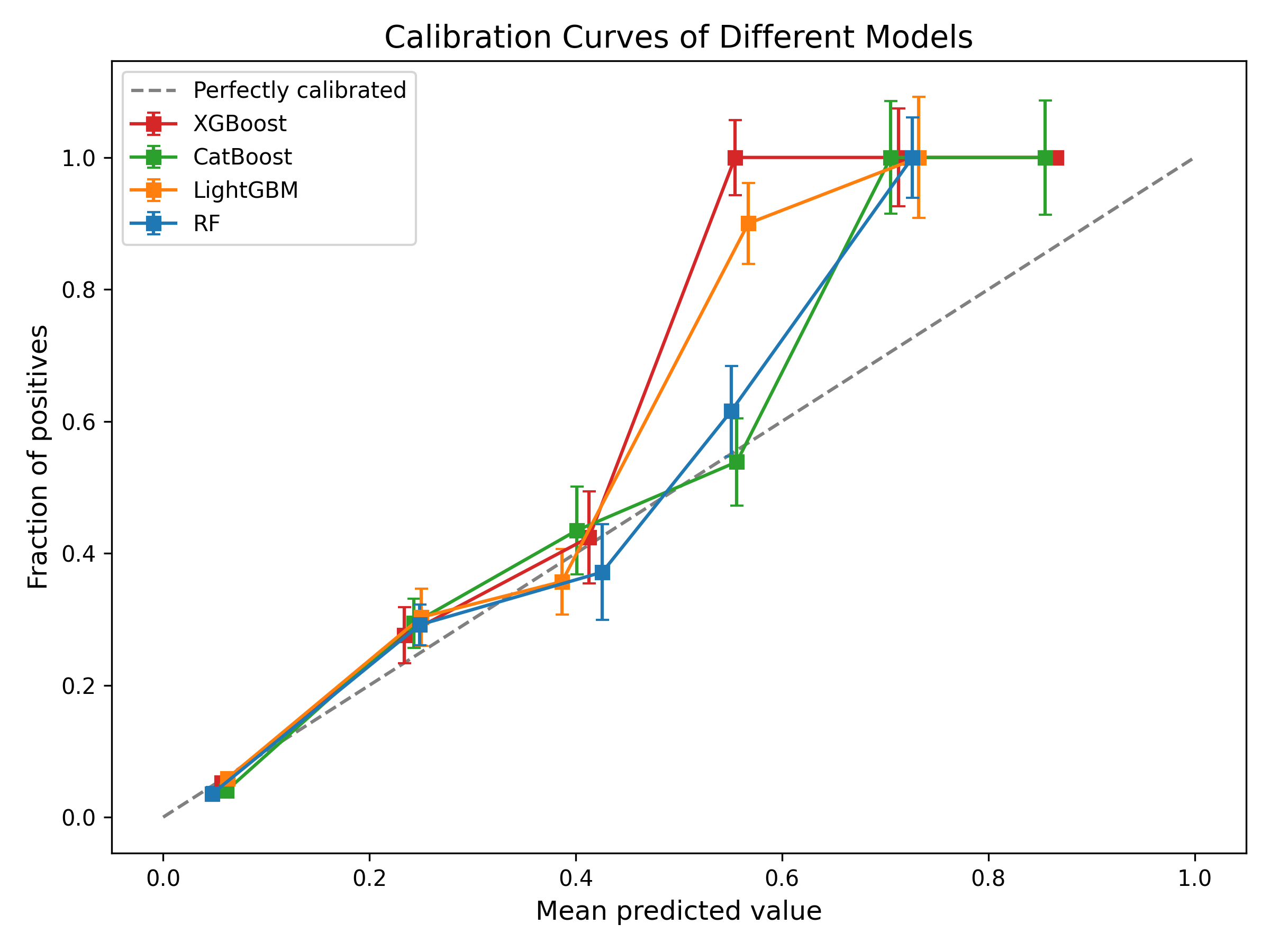

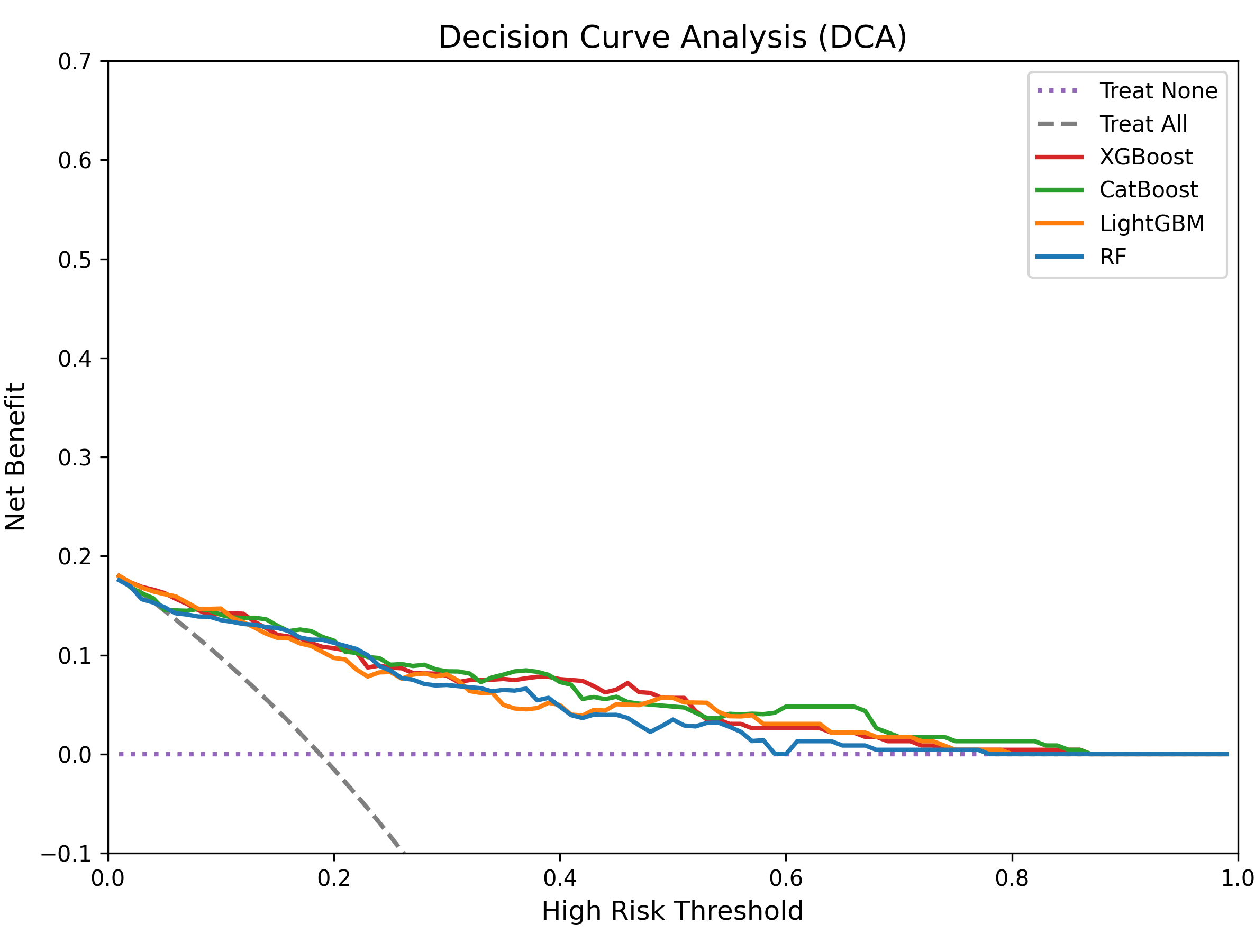

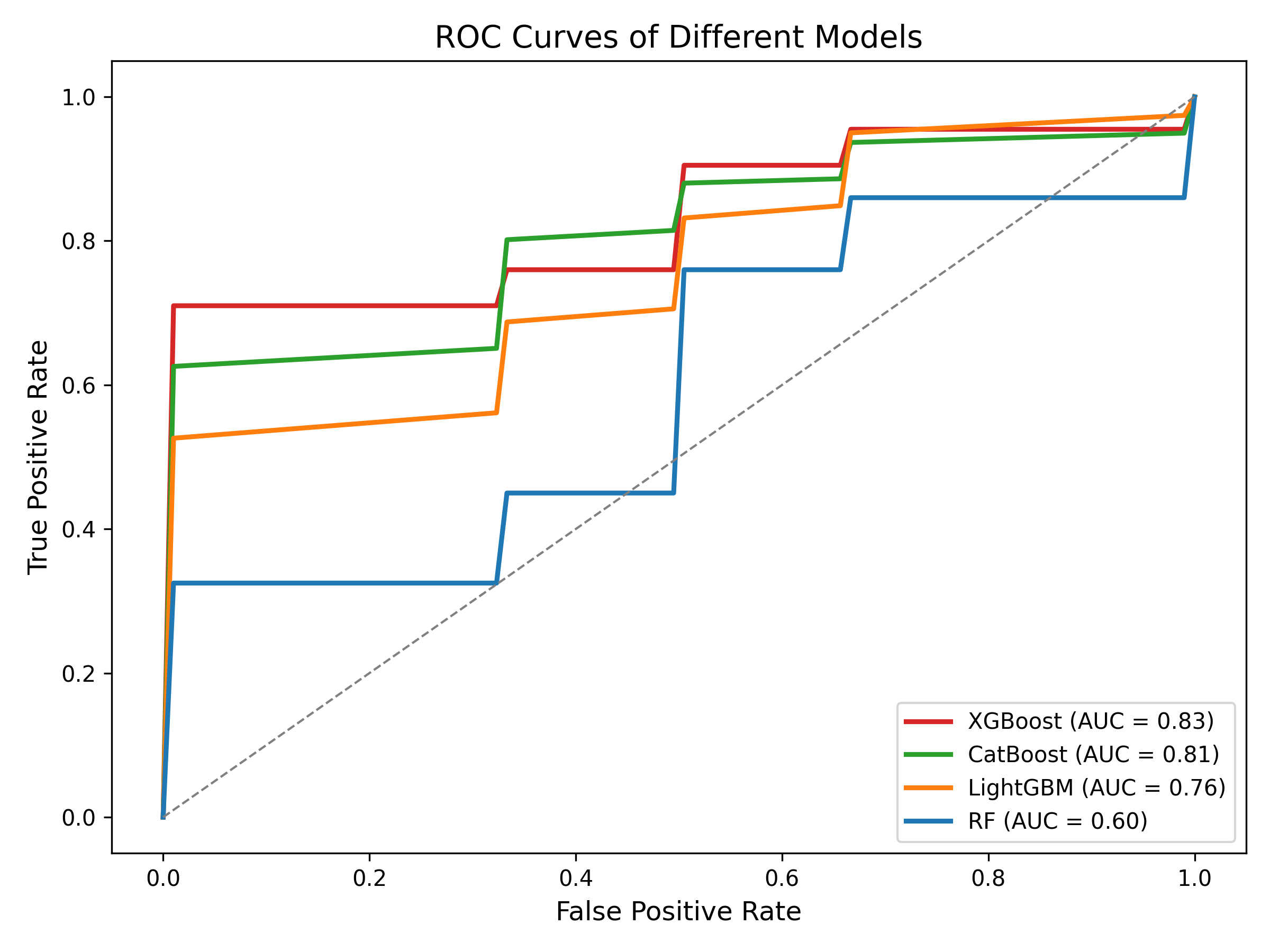

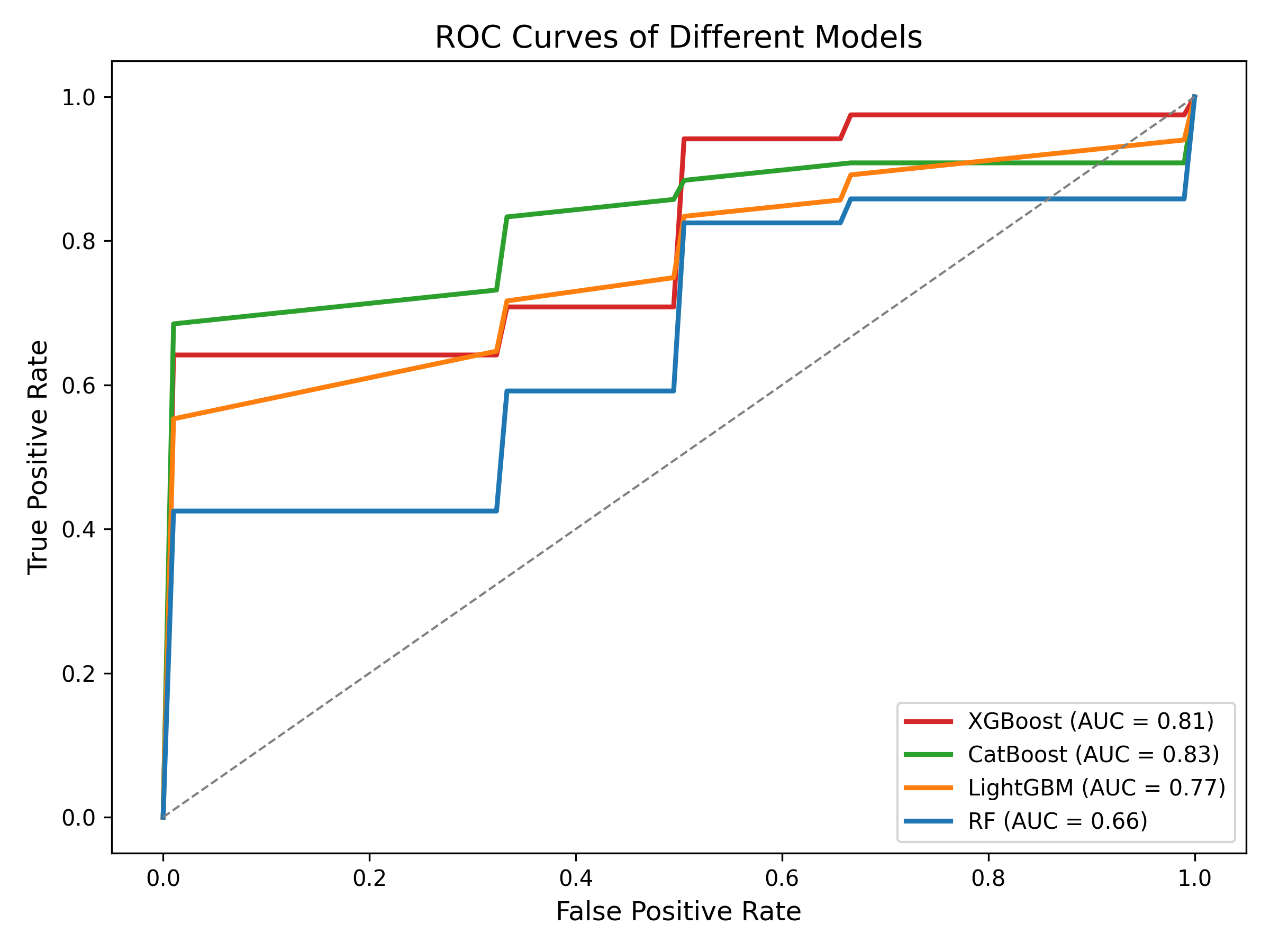

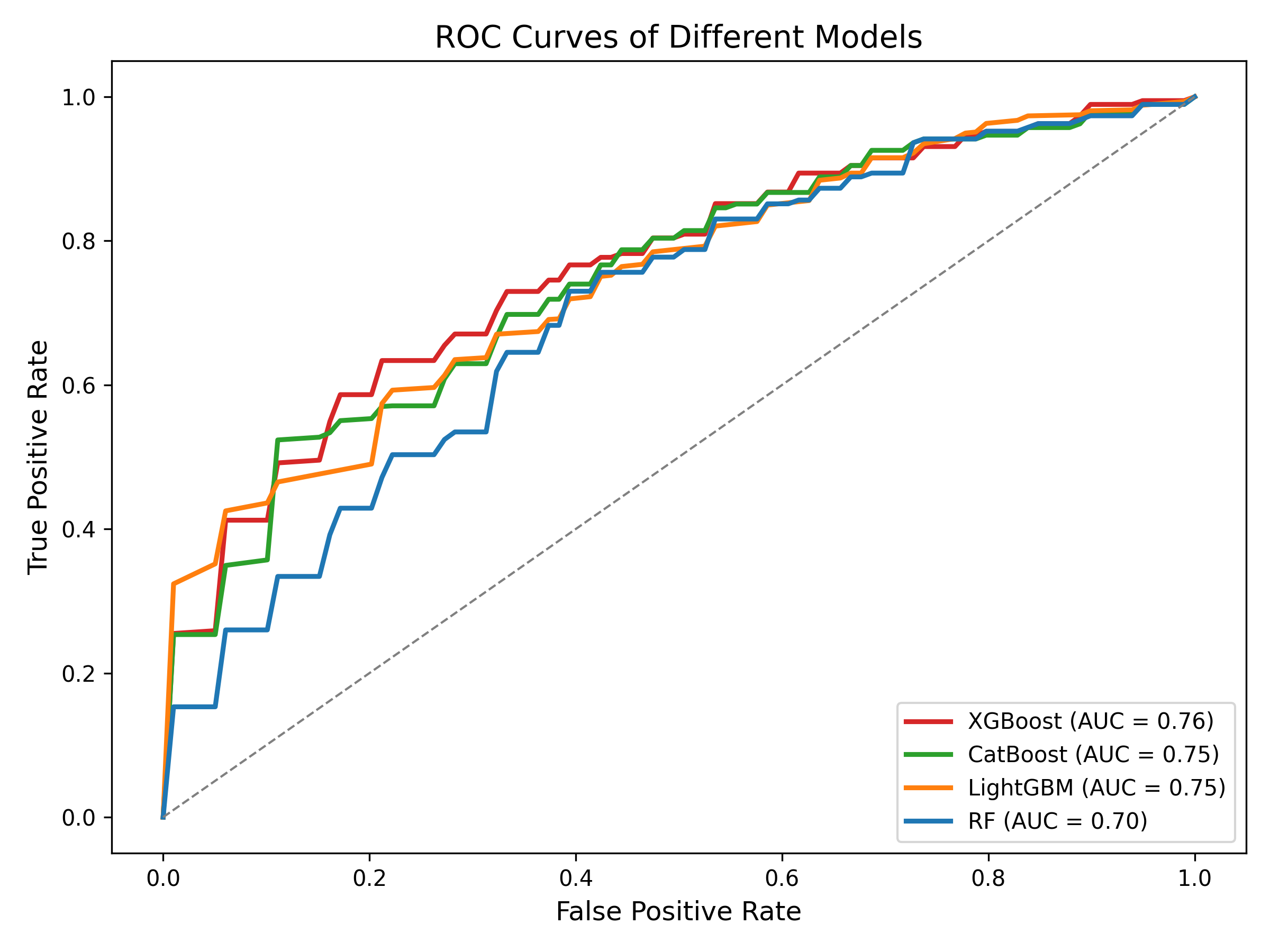

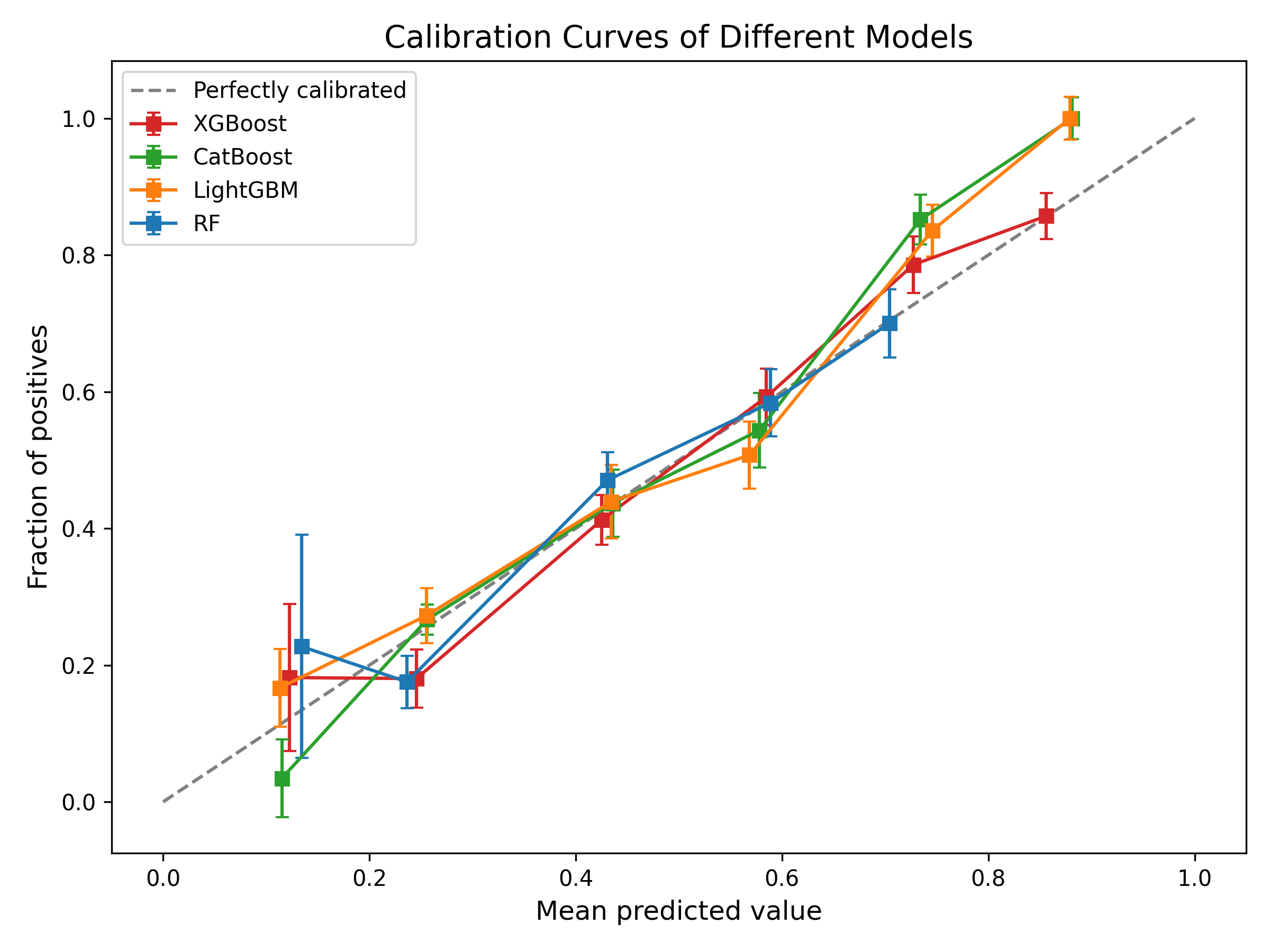

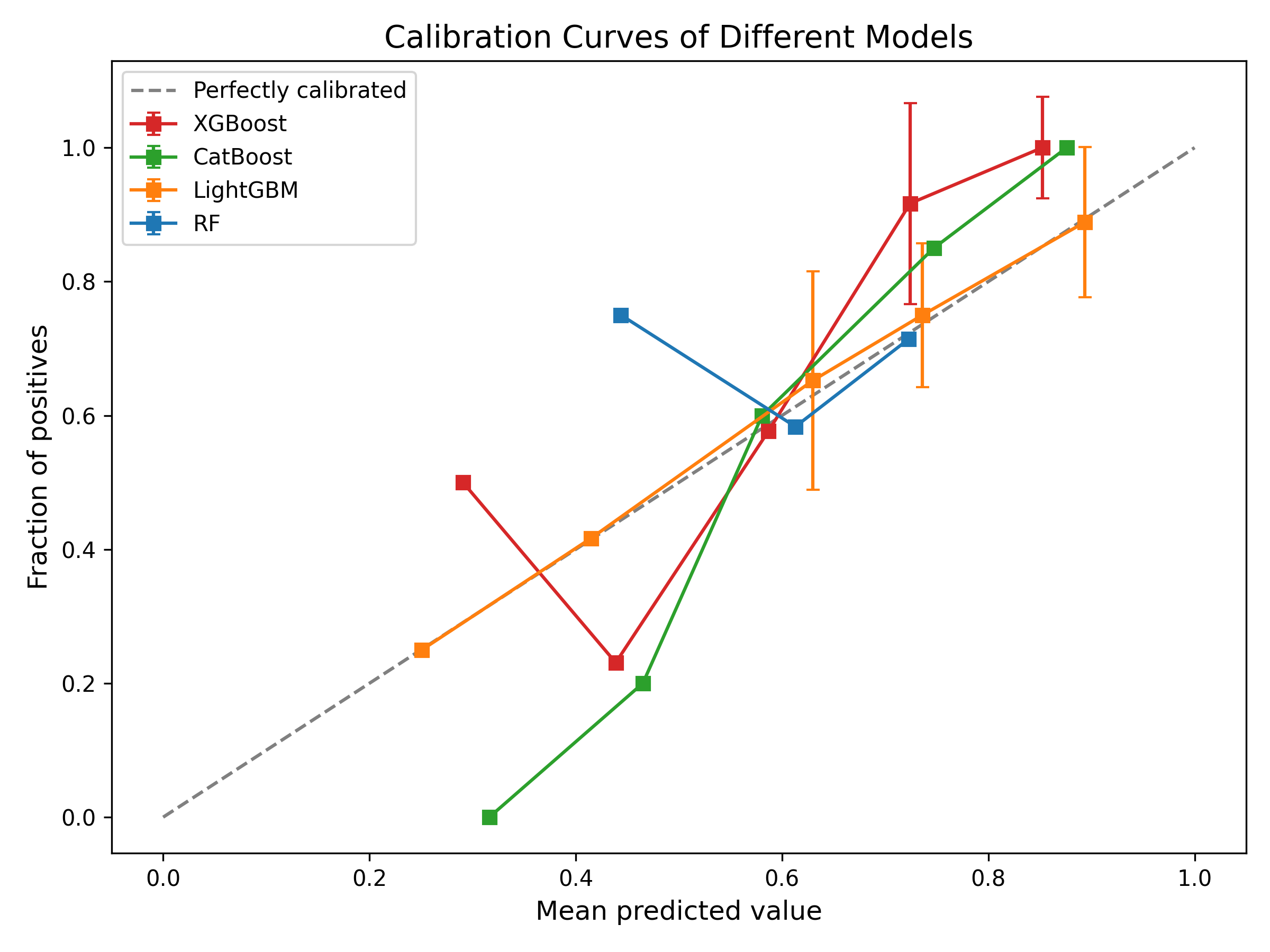

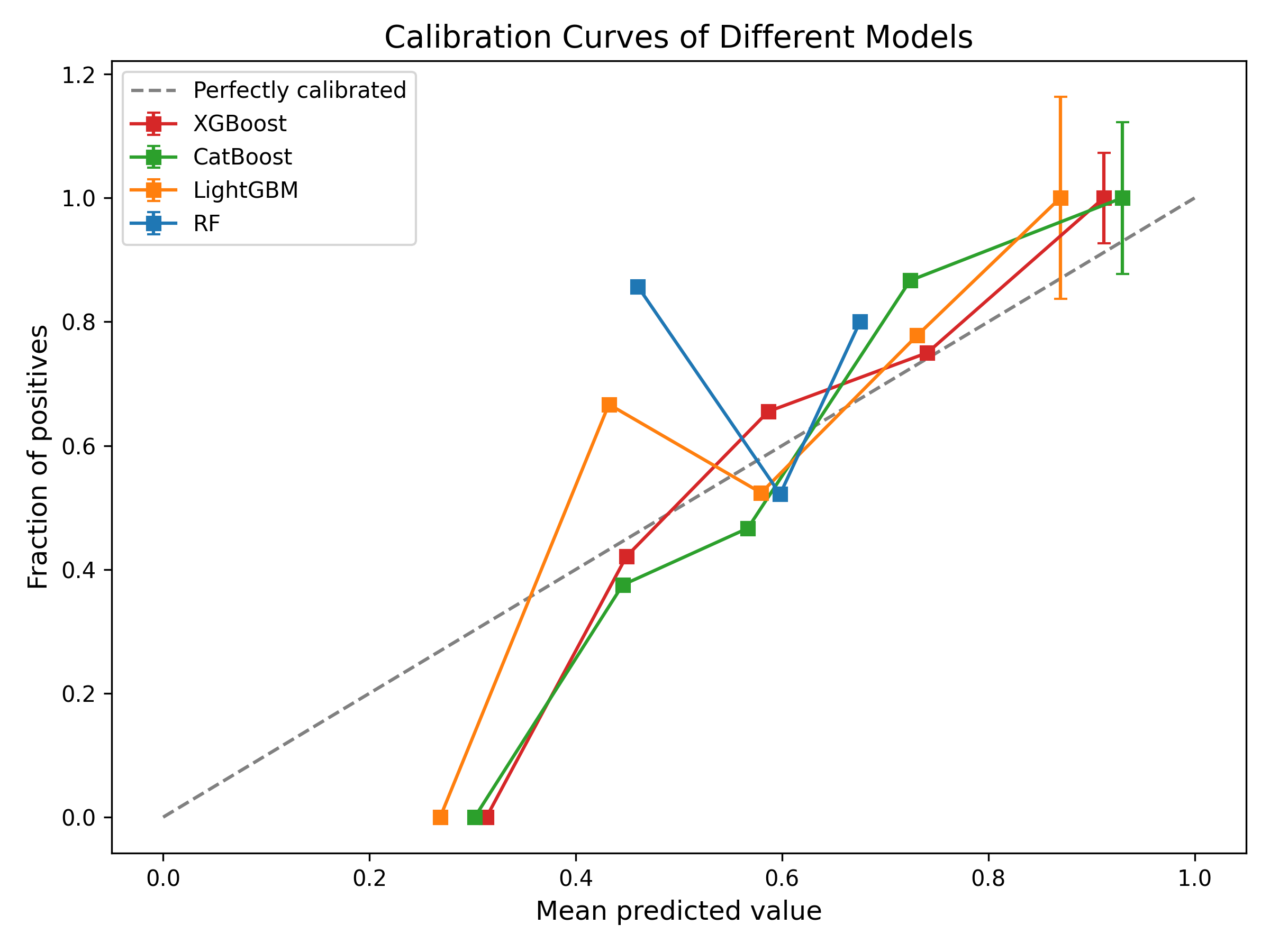

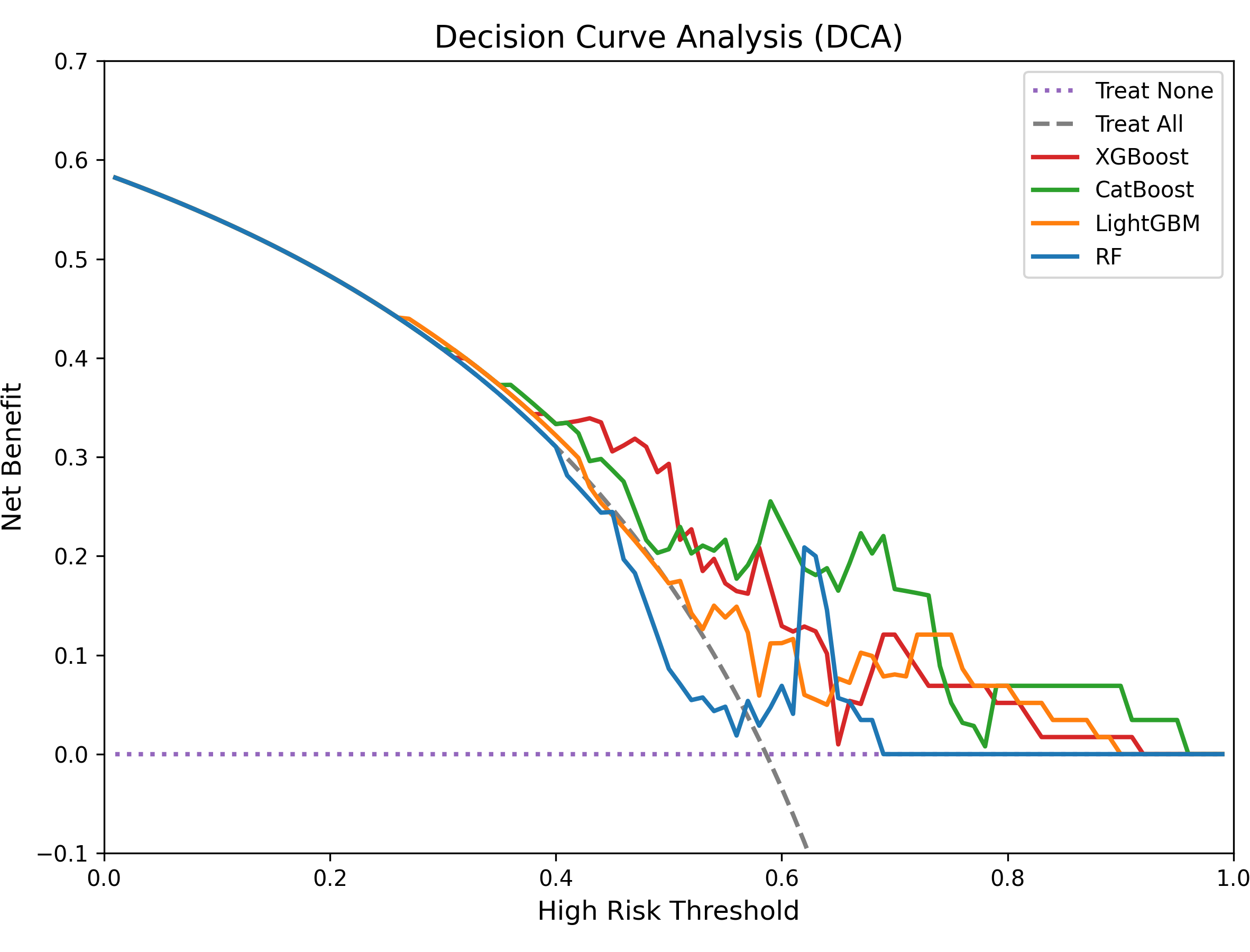

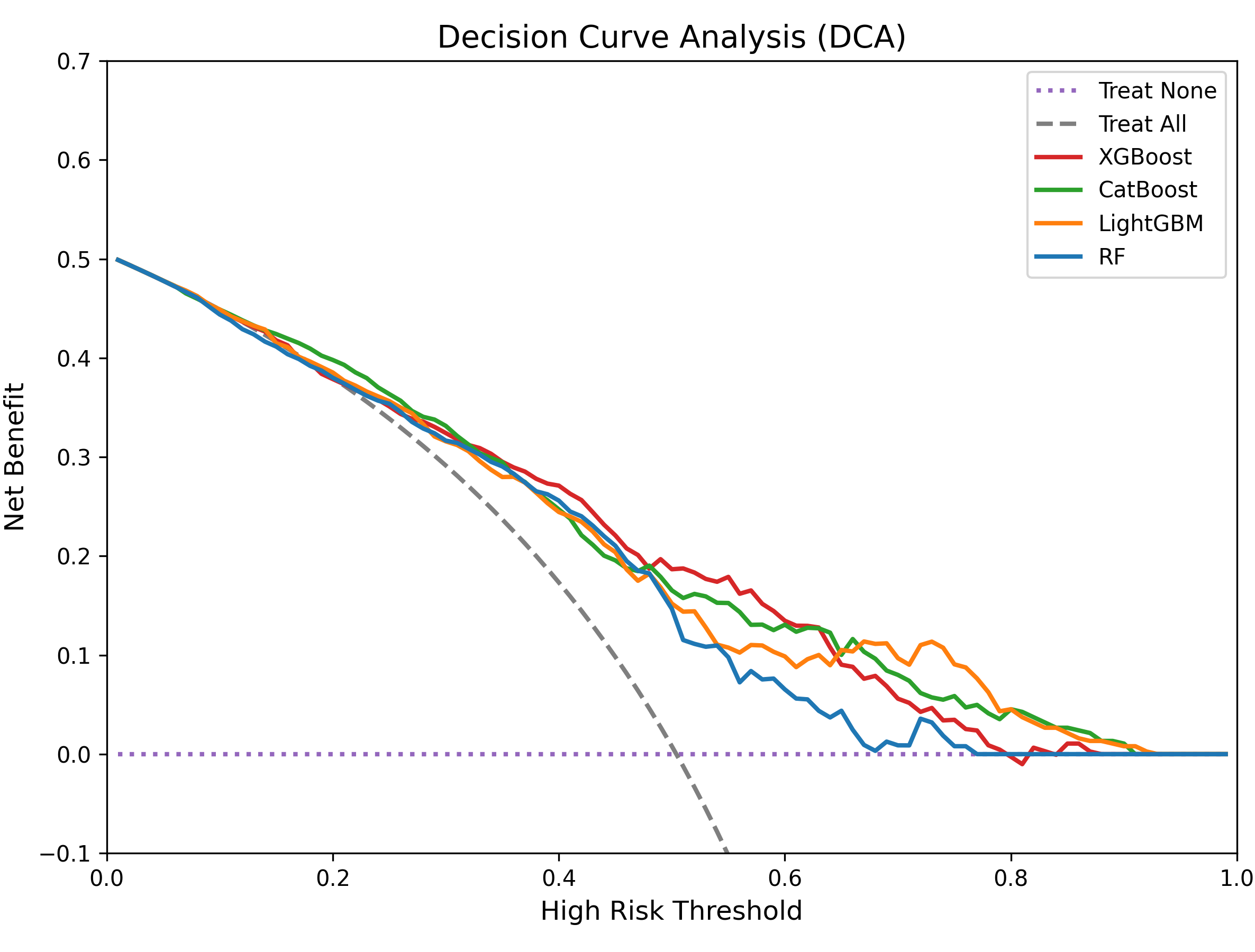

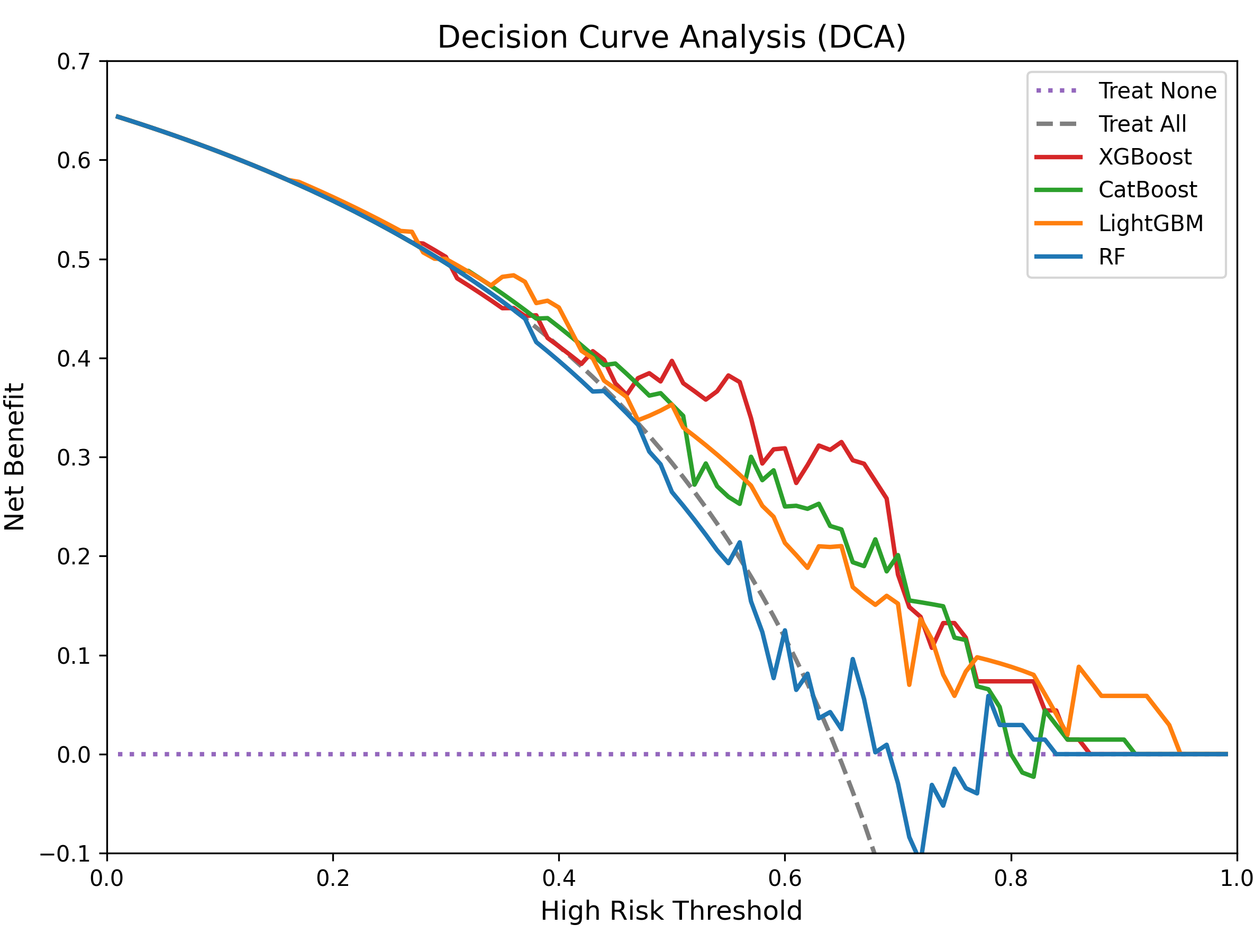


From left to right, the columns represent the four prediction tasks: pG, pN, rG, and rN. From top to bottom, the rows display the AUC, calibration plots, and decision DCA plots, respectively.

# Figure S2 Plots of each task using features selected by RFE


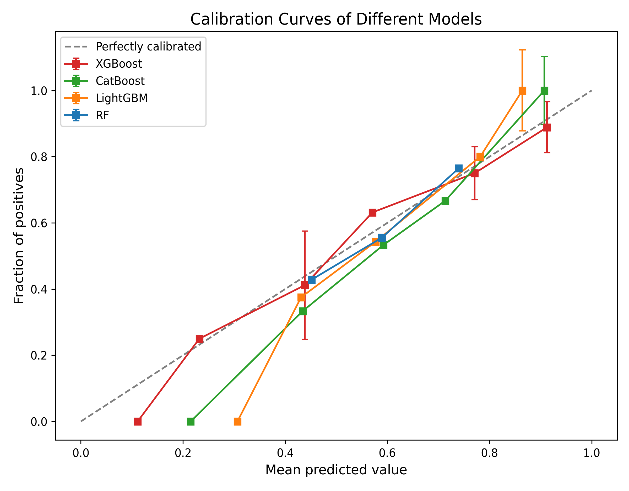

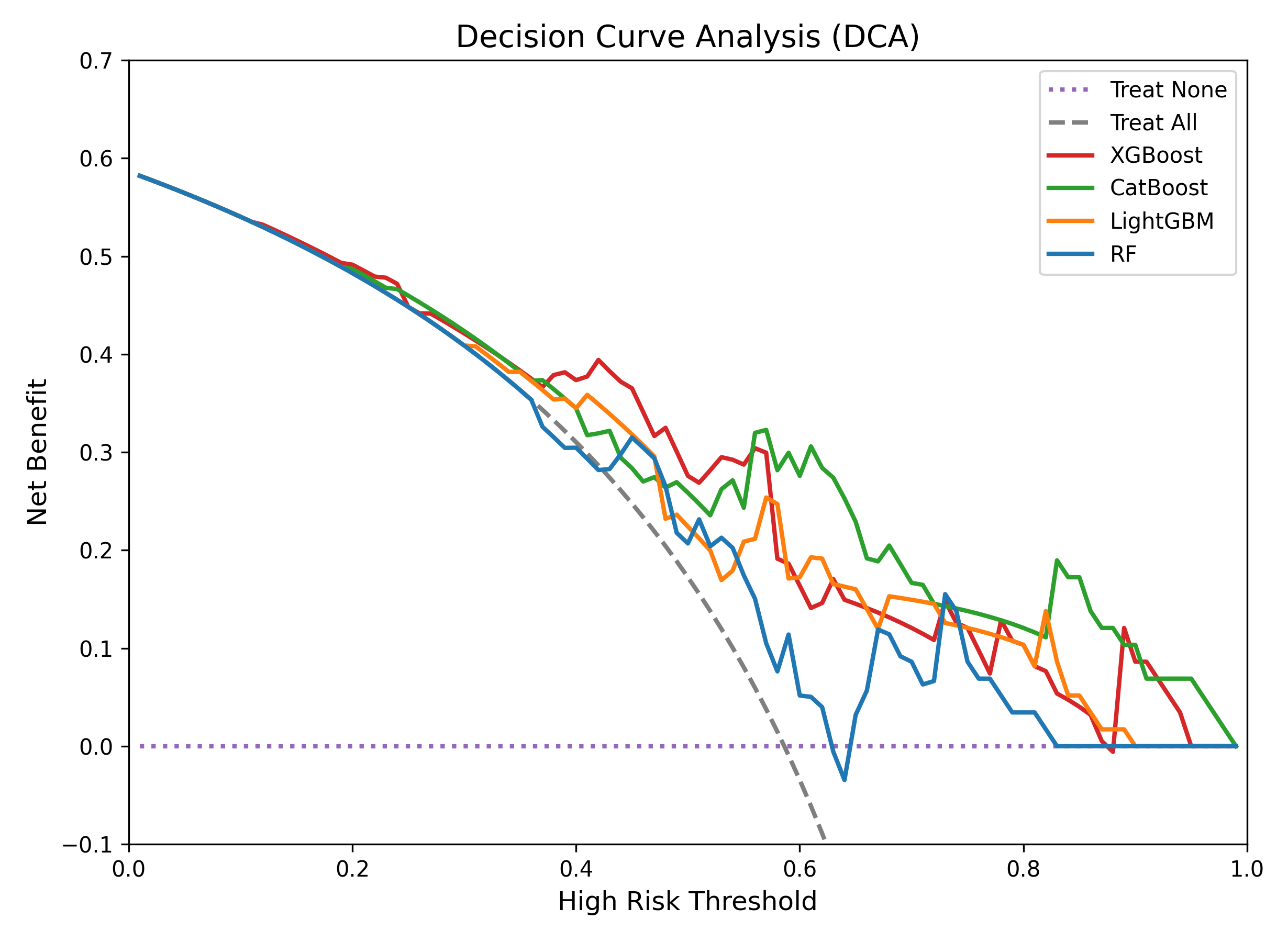

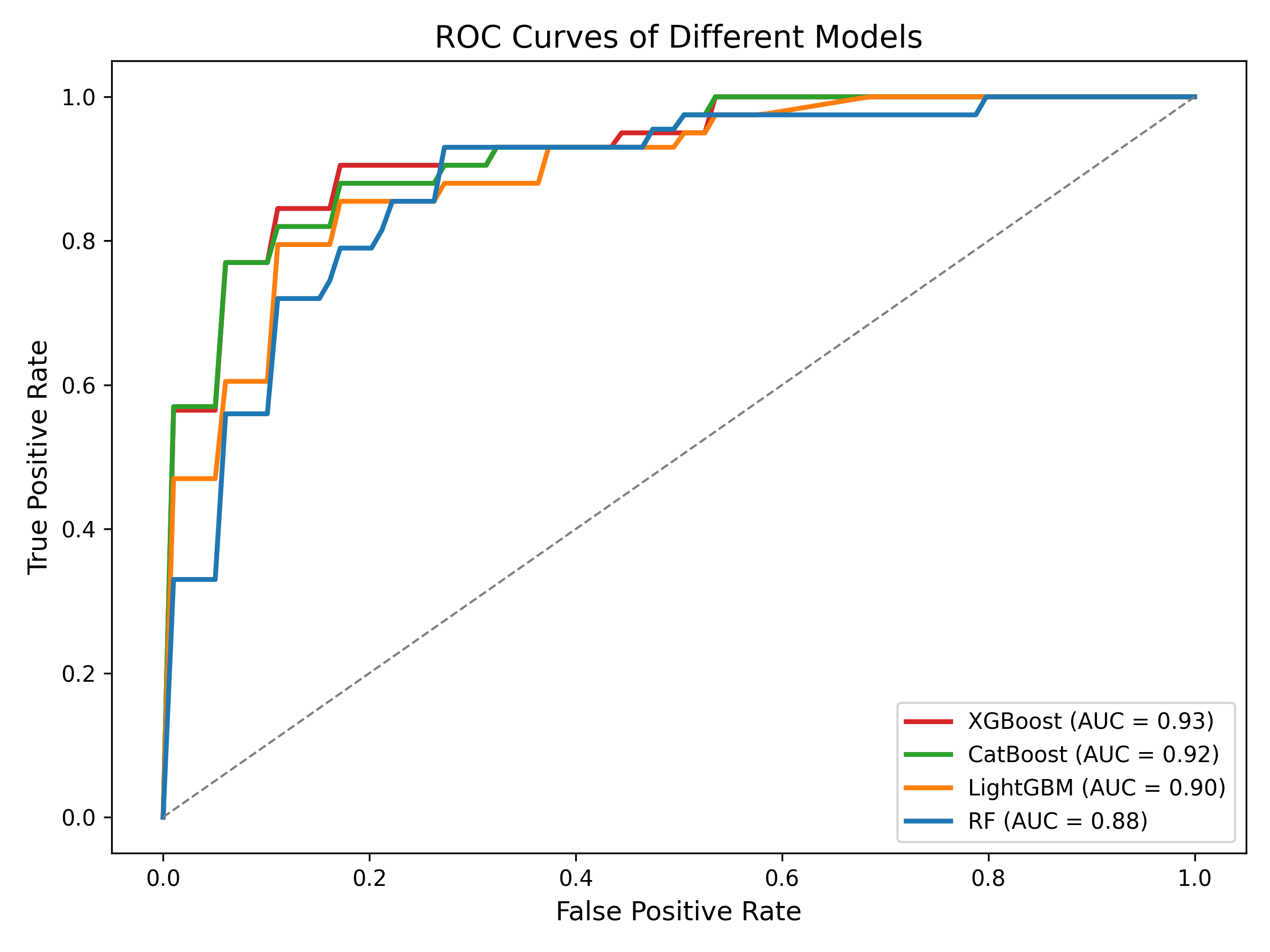

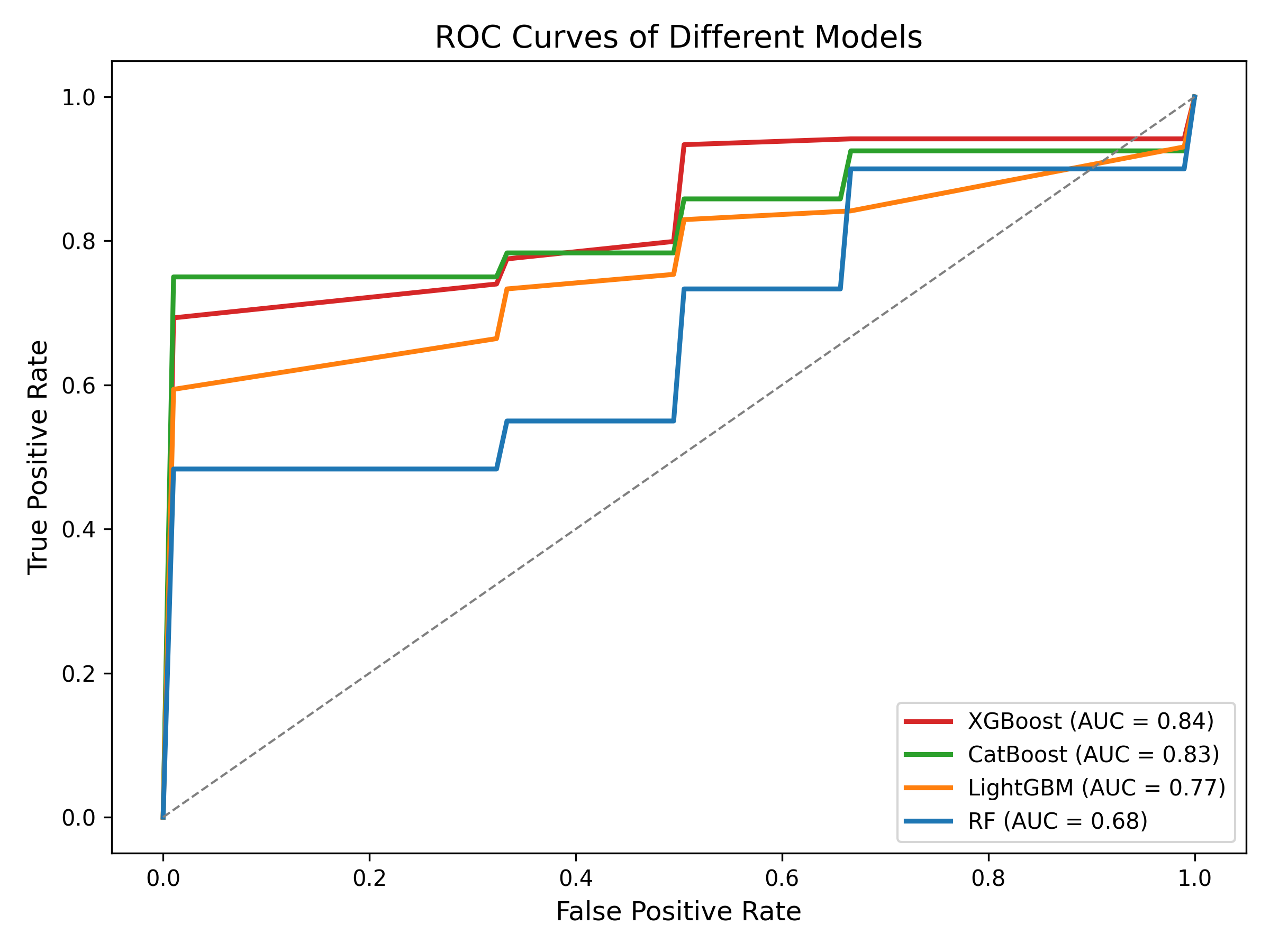

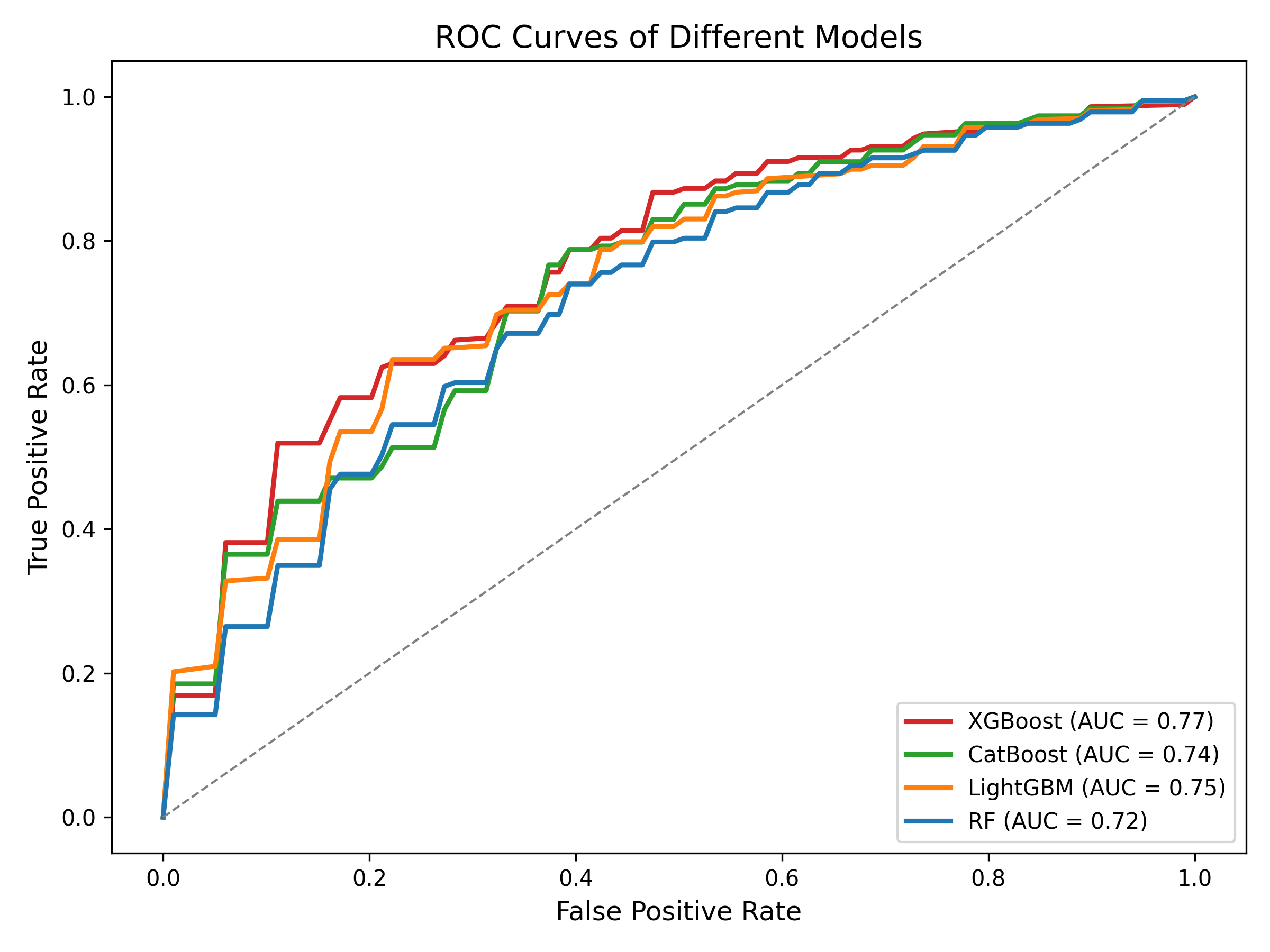

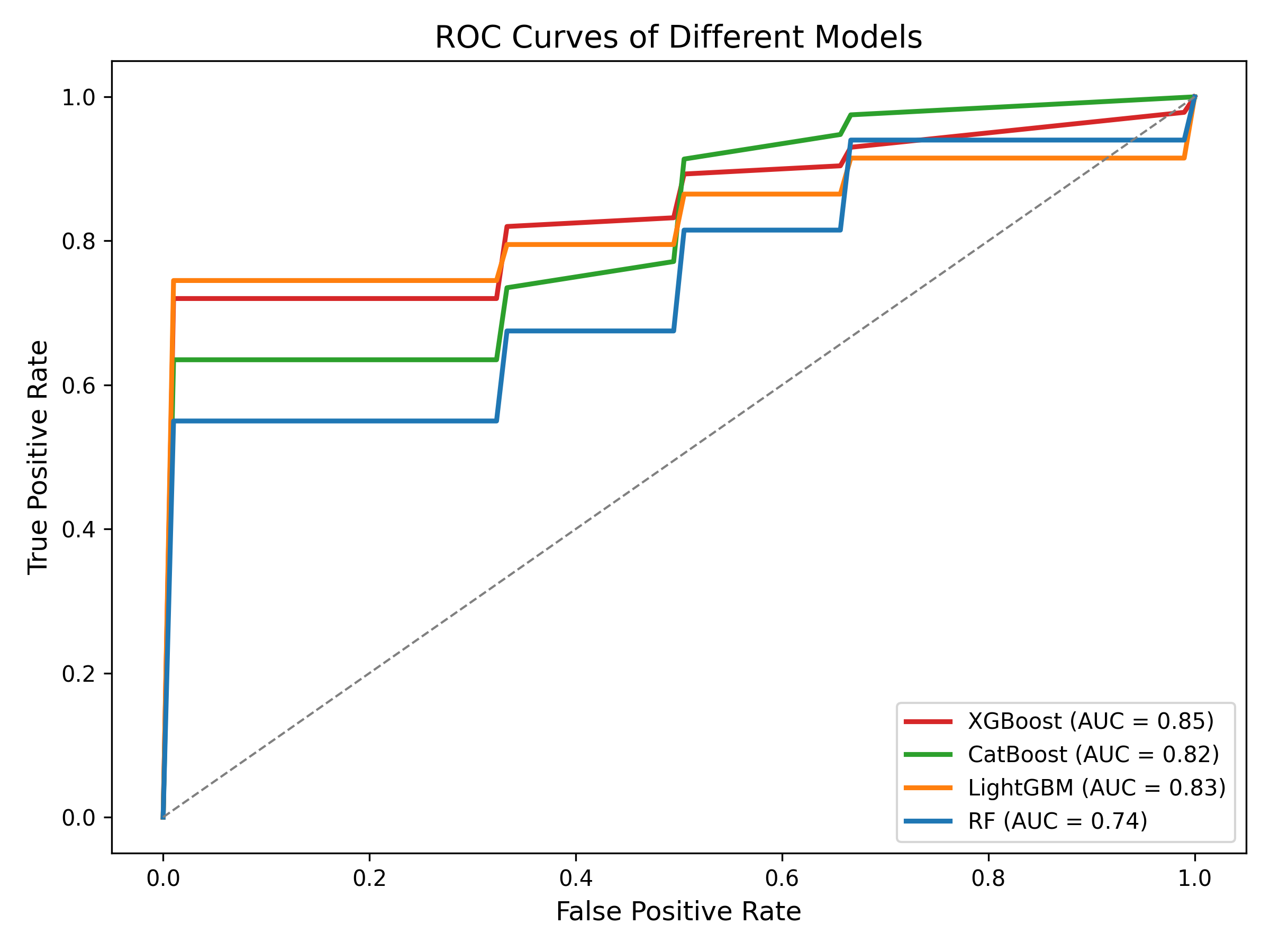

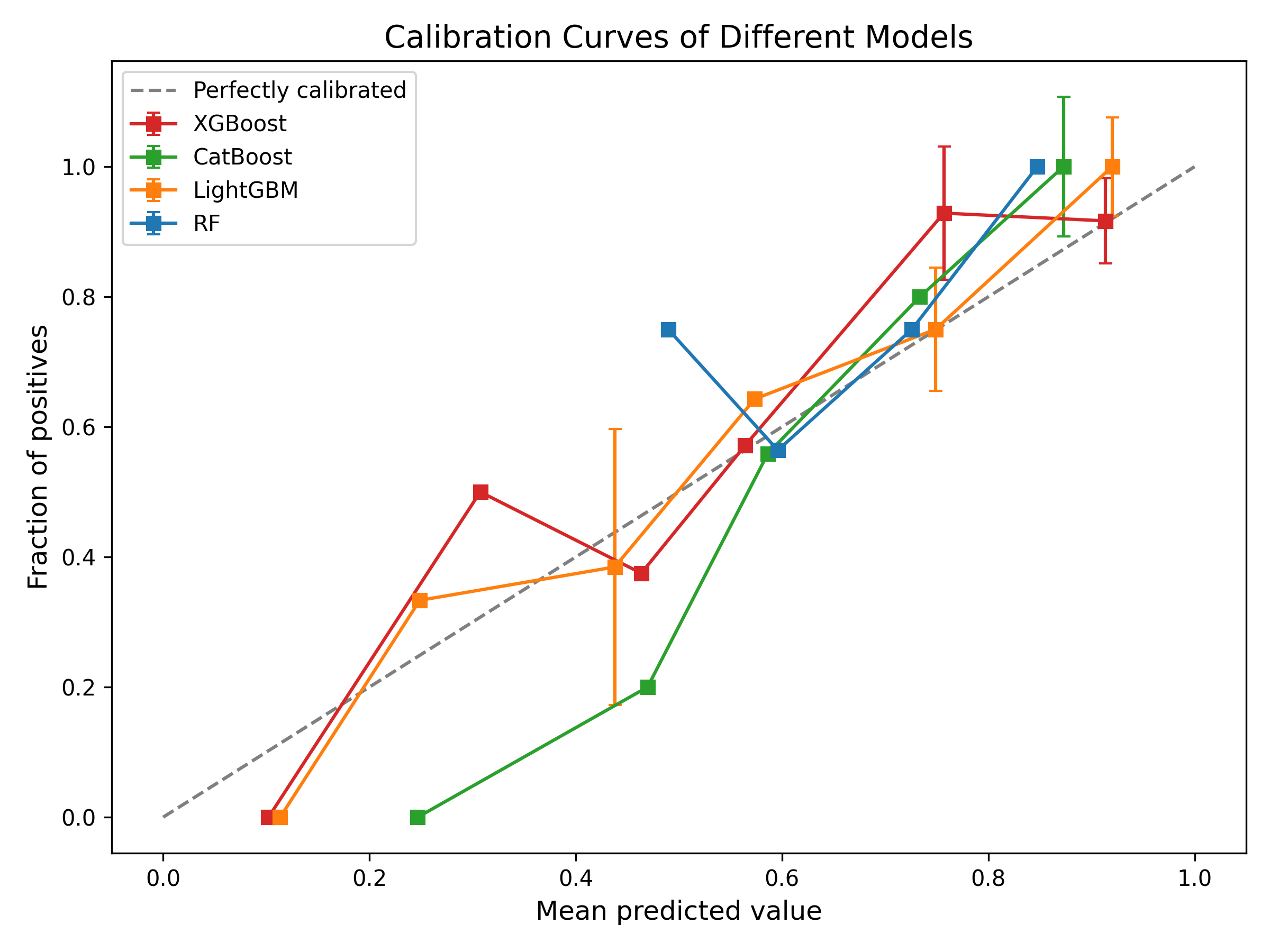

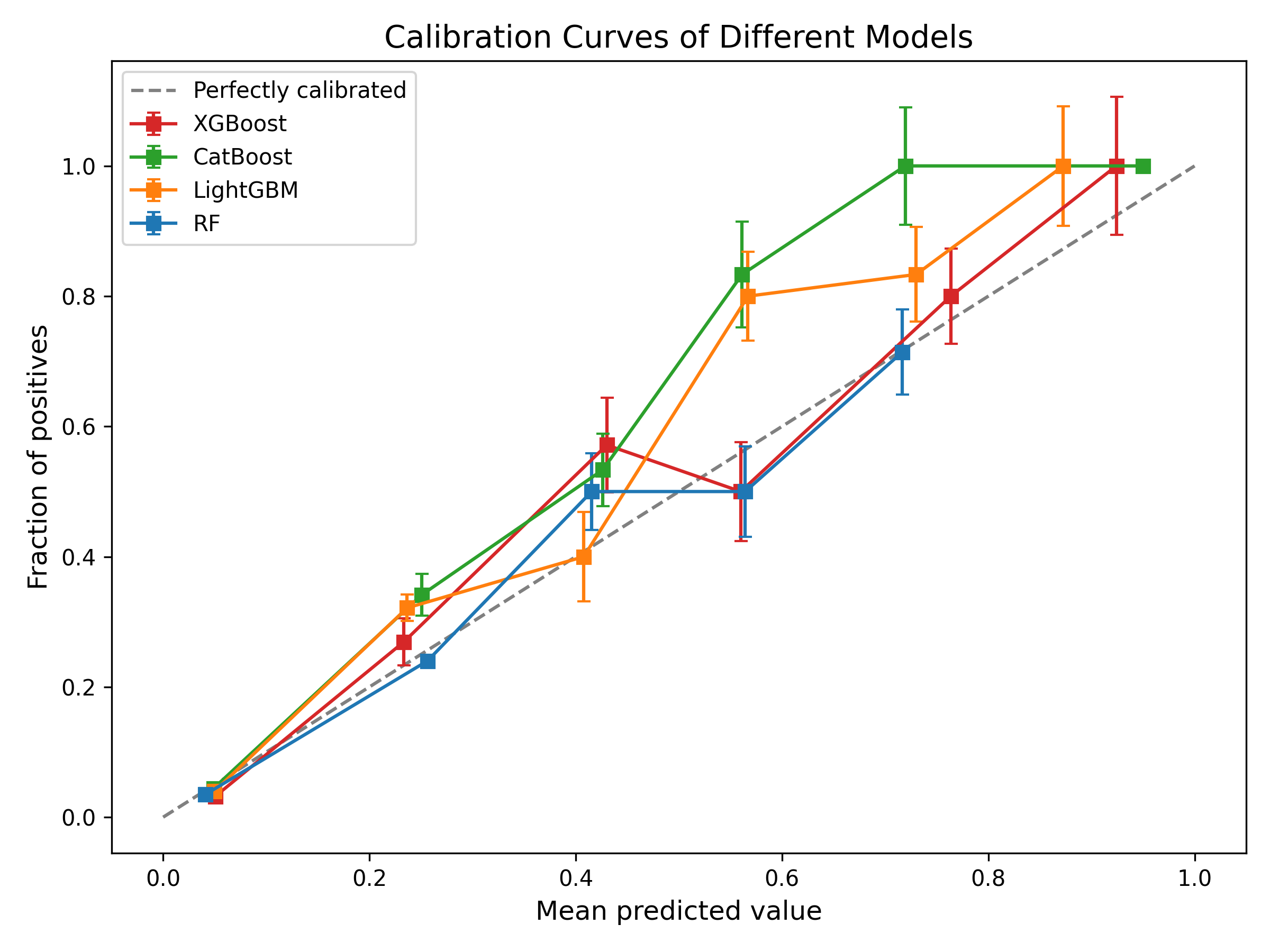

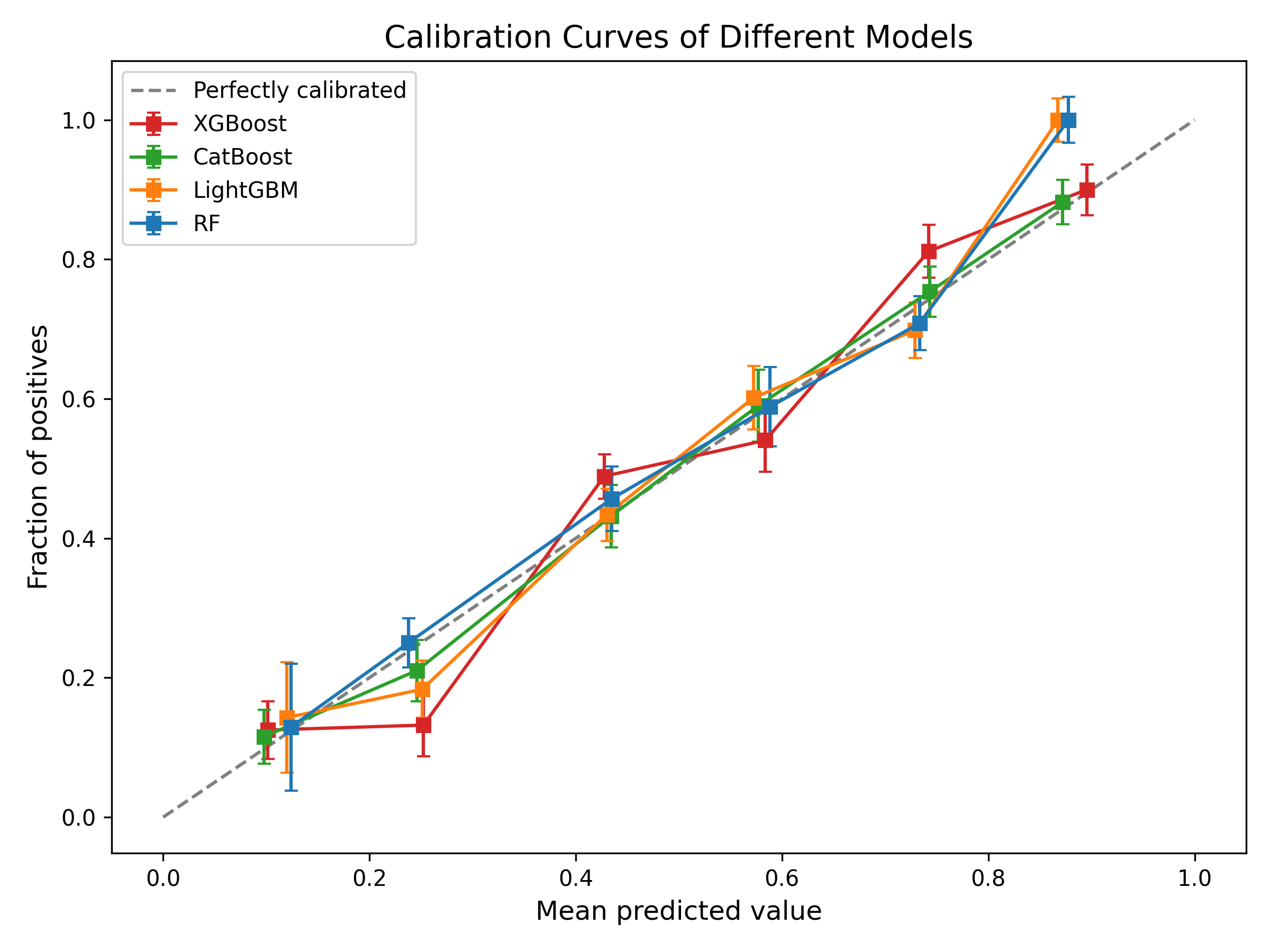

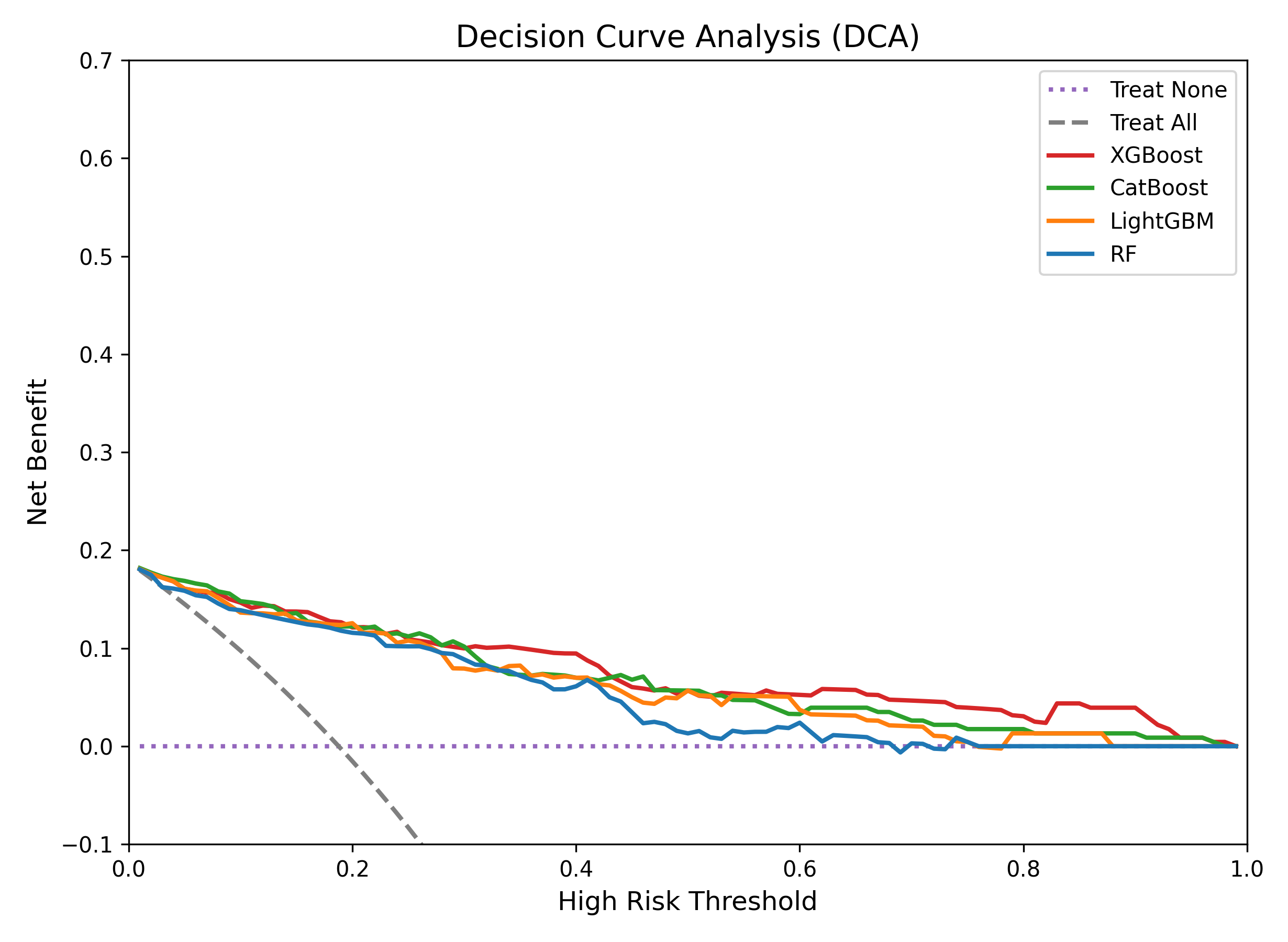

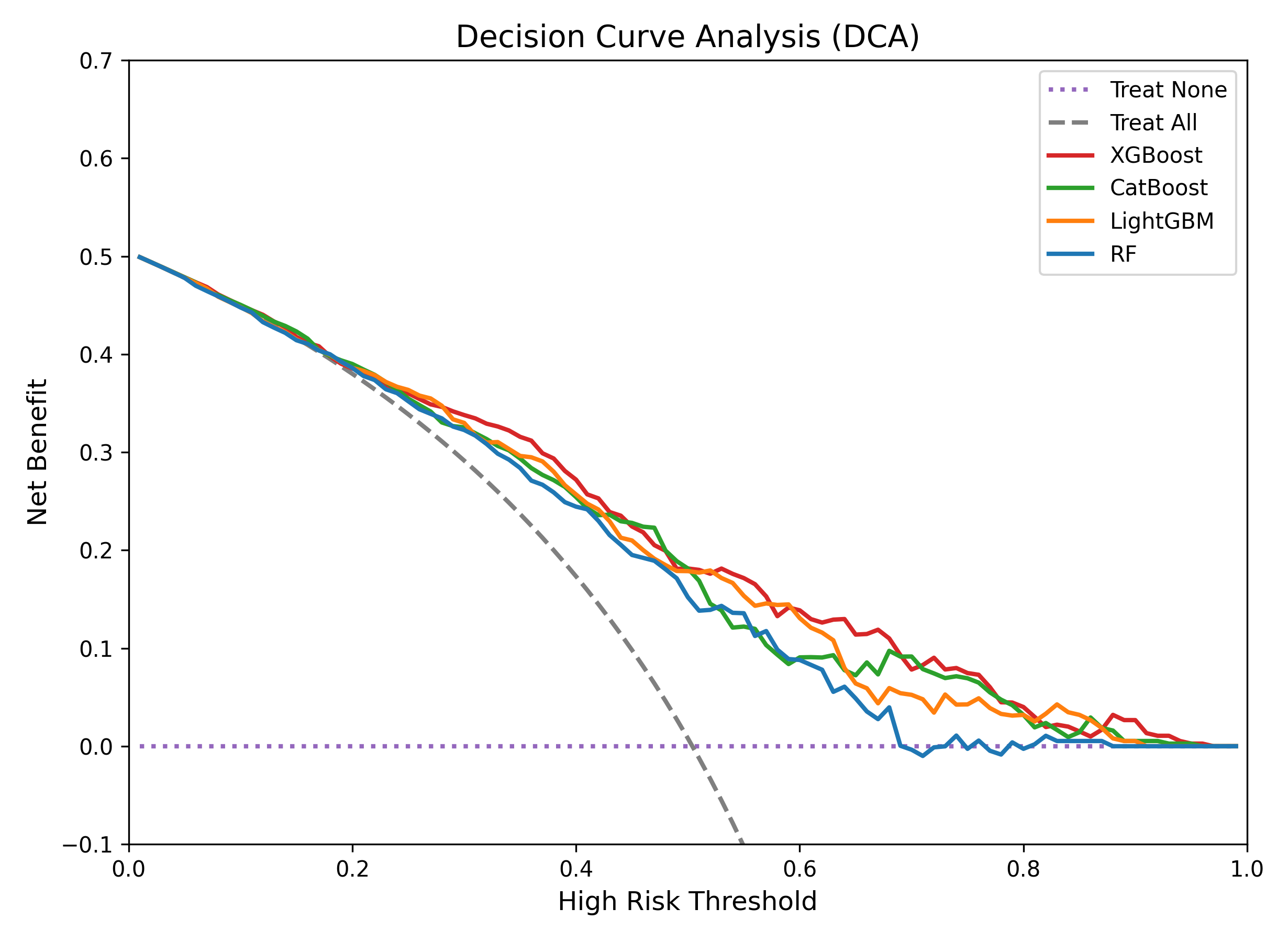

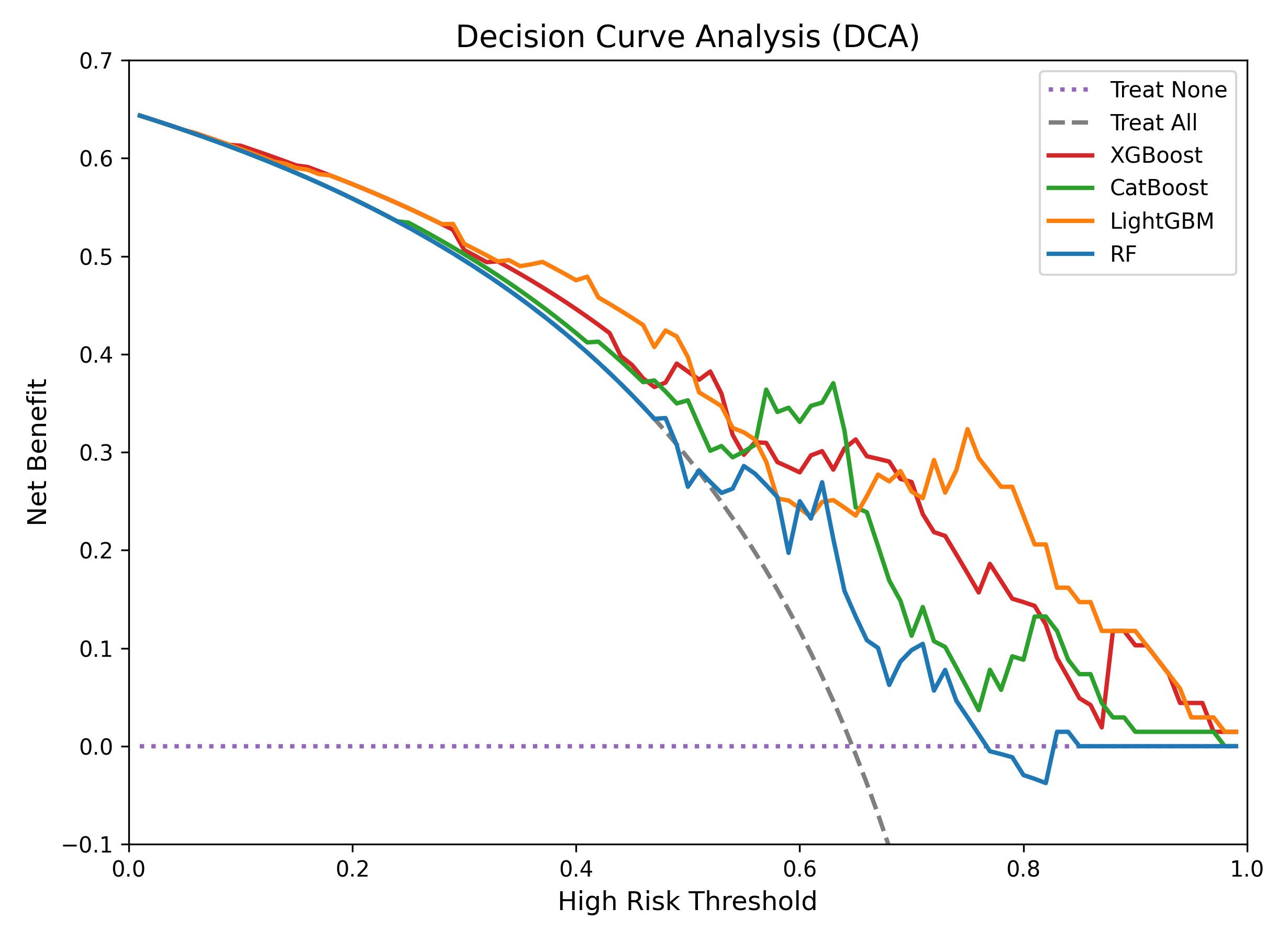


From left to right, the columns represent the four prediction tasks: pG, pN, rG, and rN. From top to bottom, the rows display the AUC, calibration plots, and decision DCA plots, respectively.

# Figure S3 Plots of each task using features selected by LASSO.


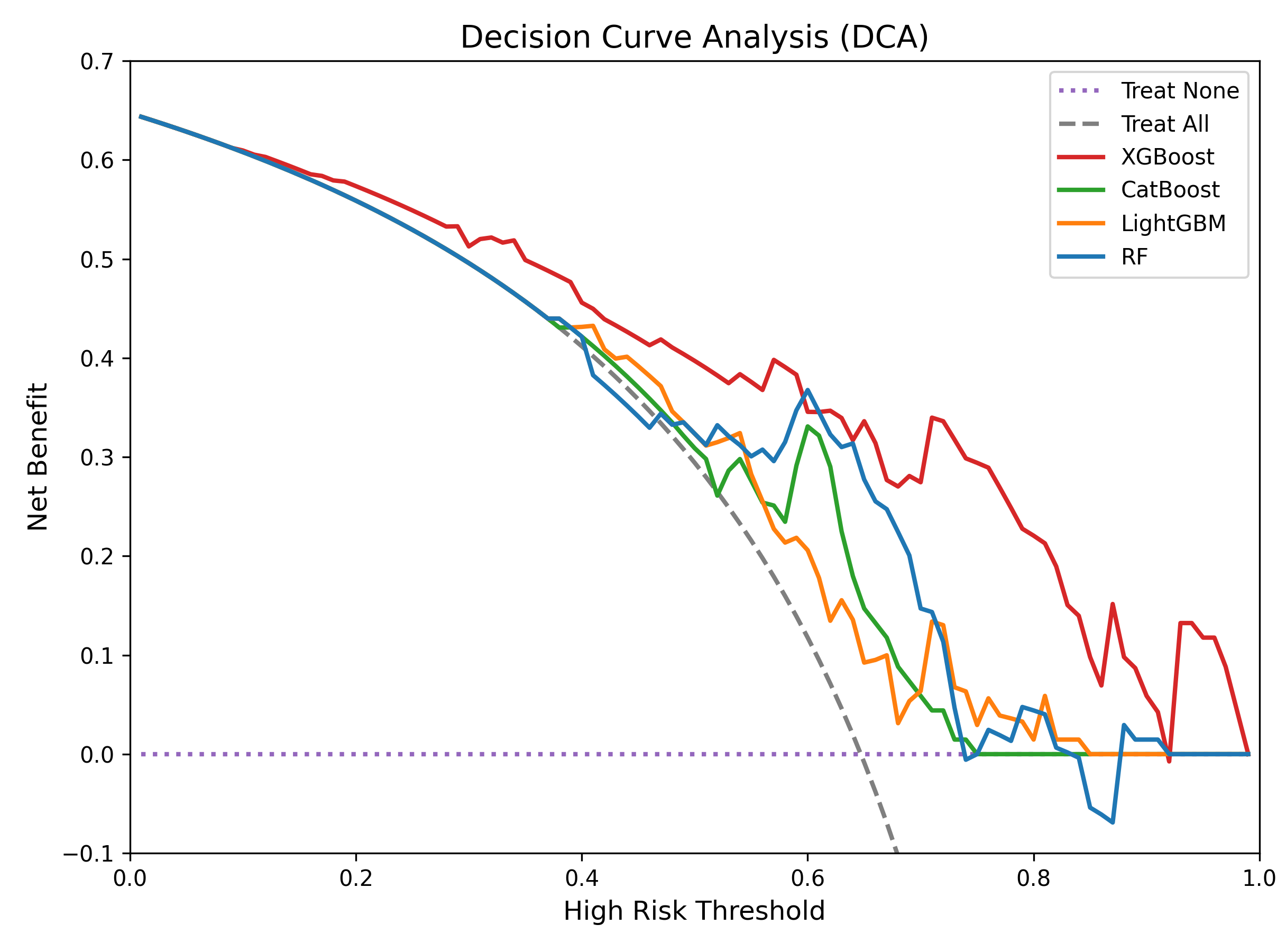

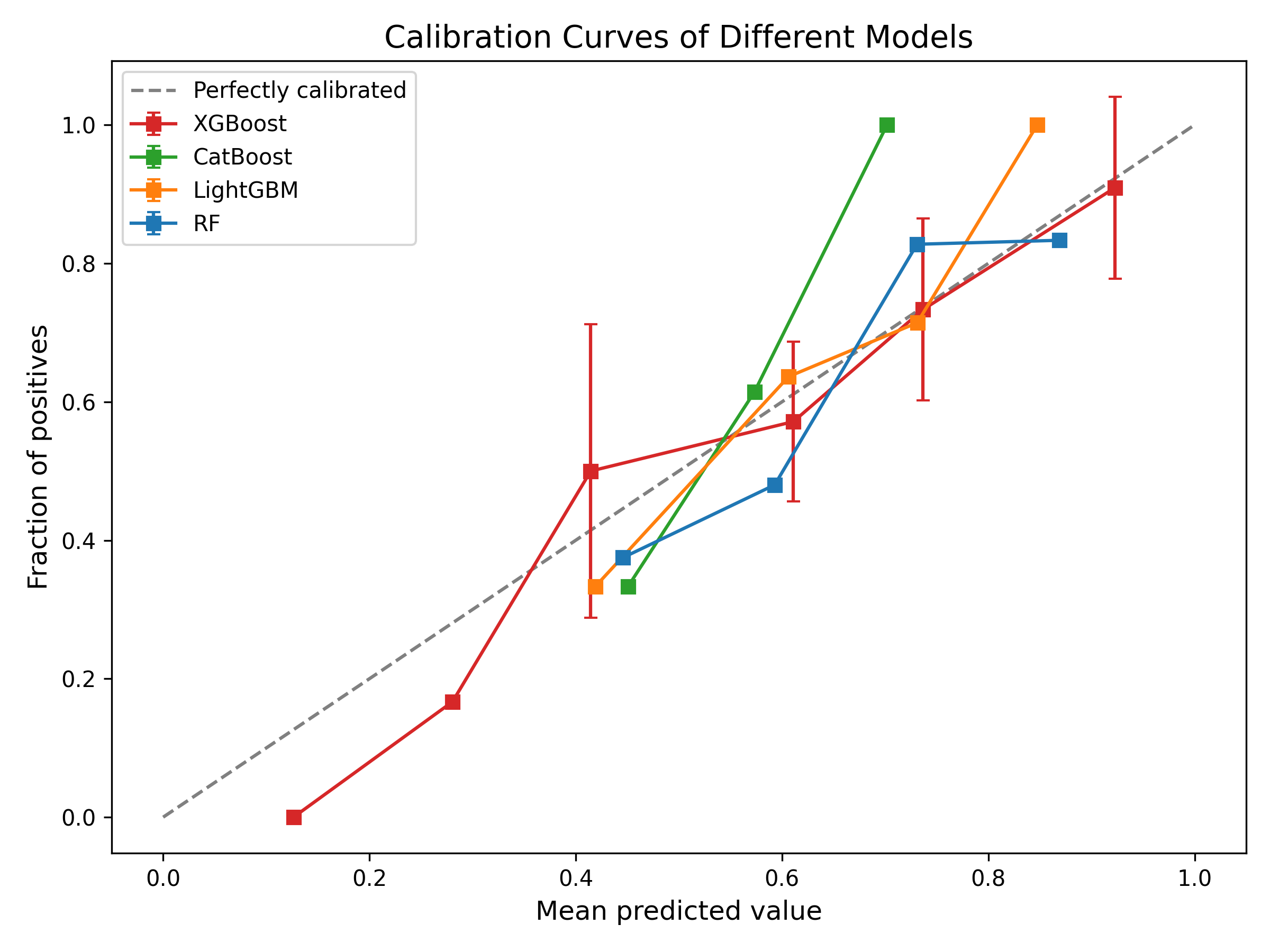

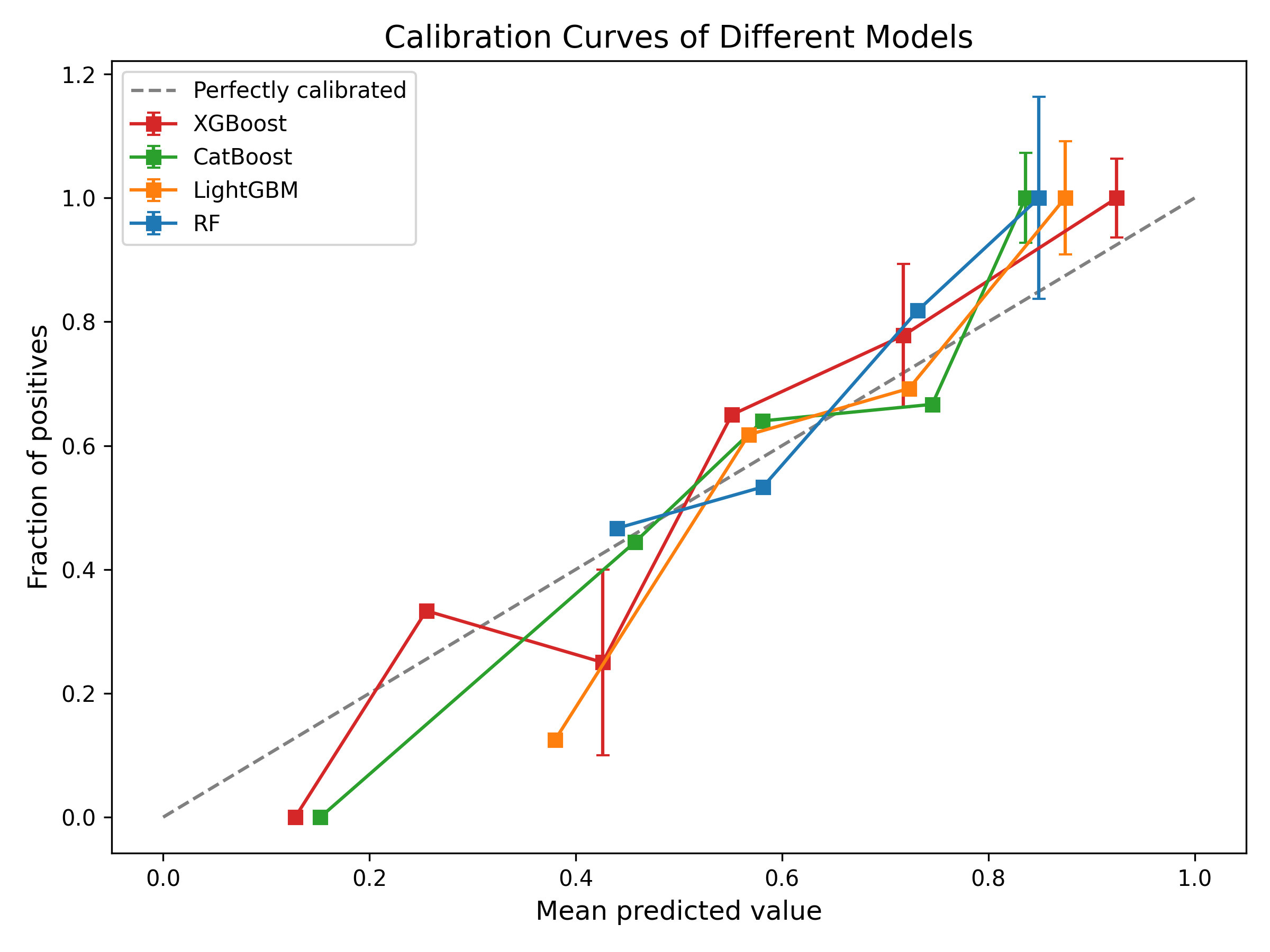

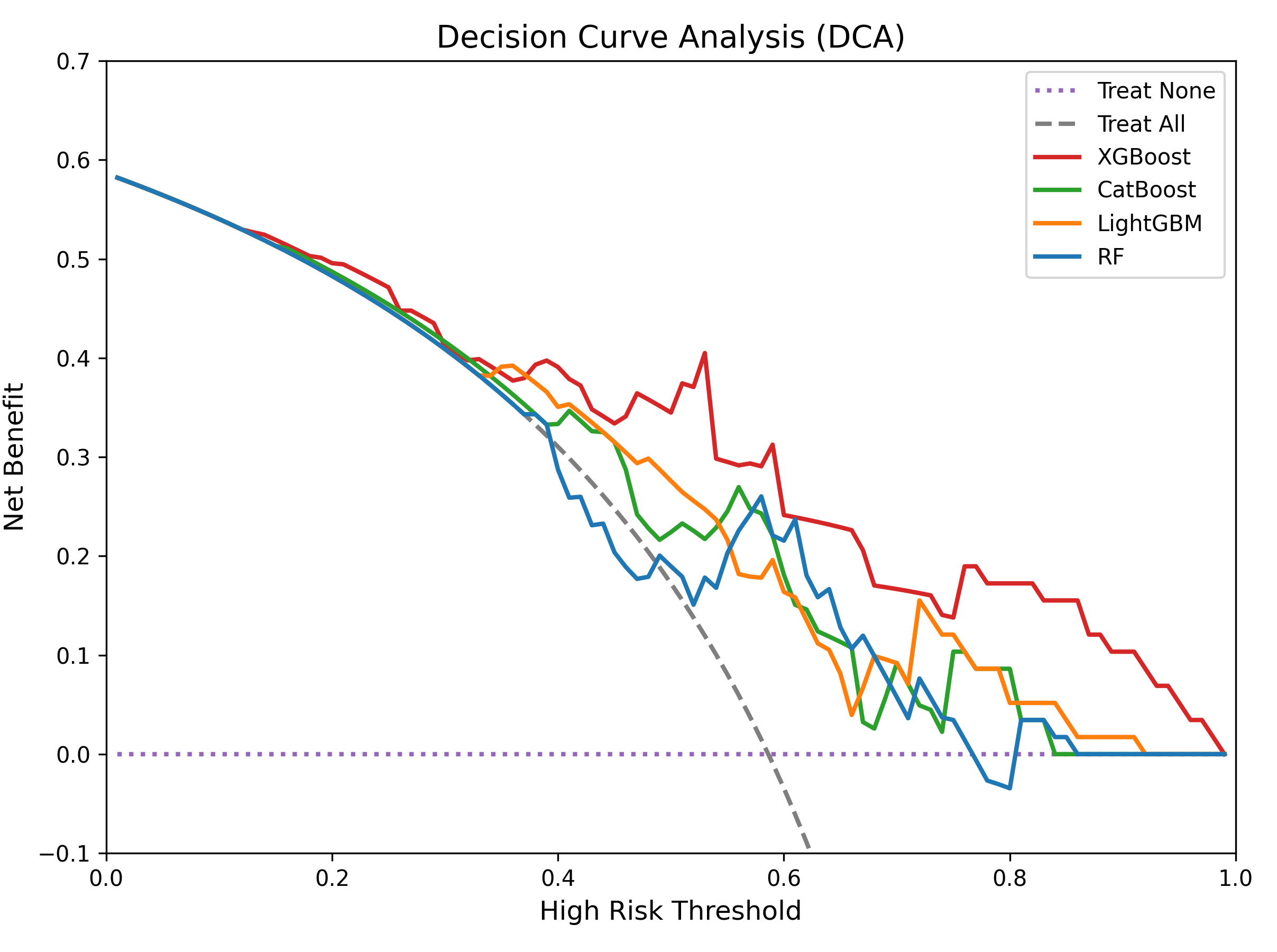

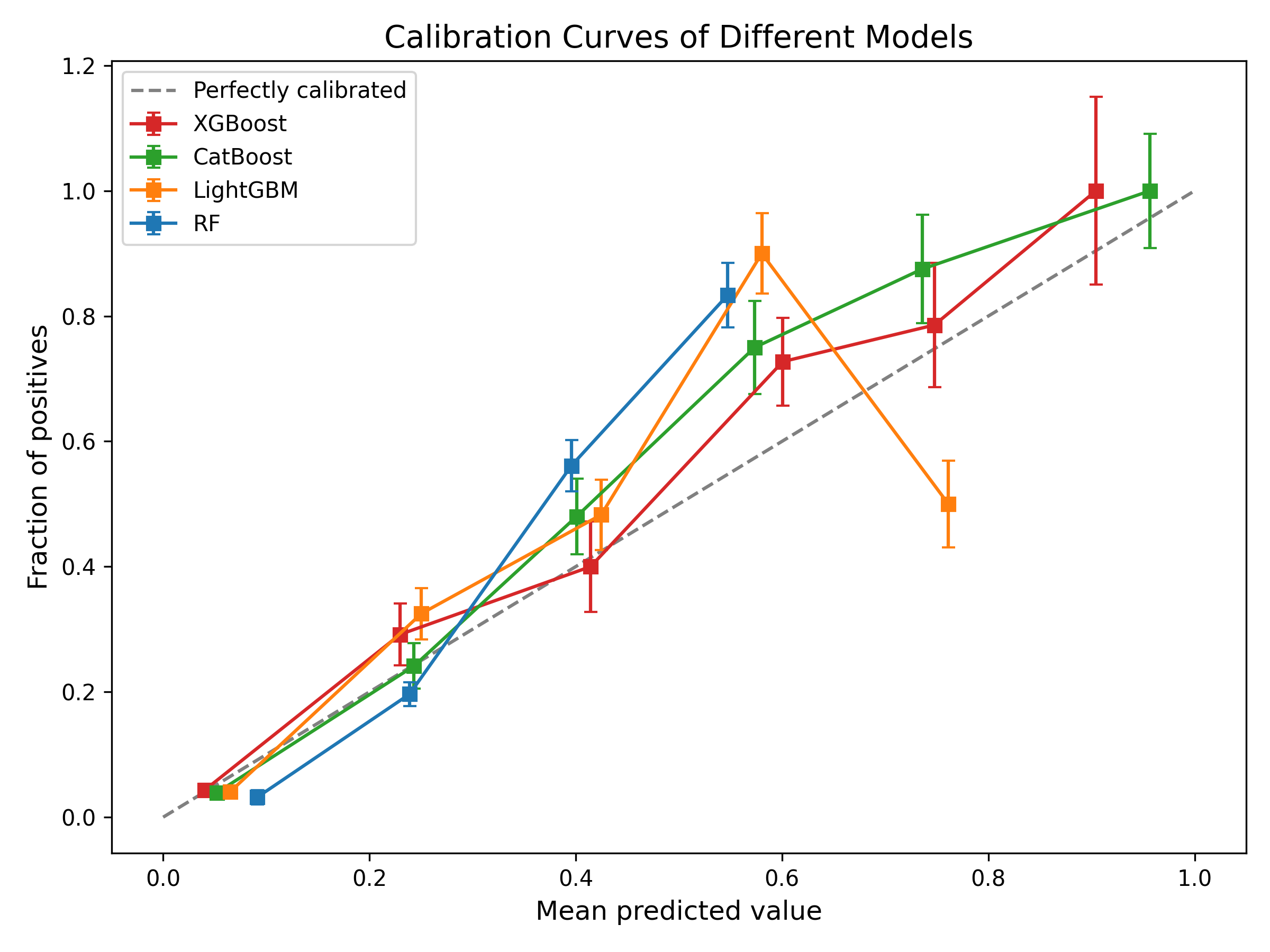

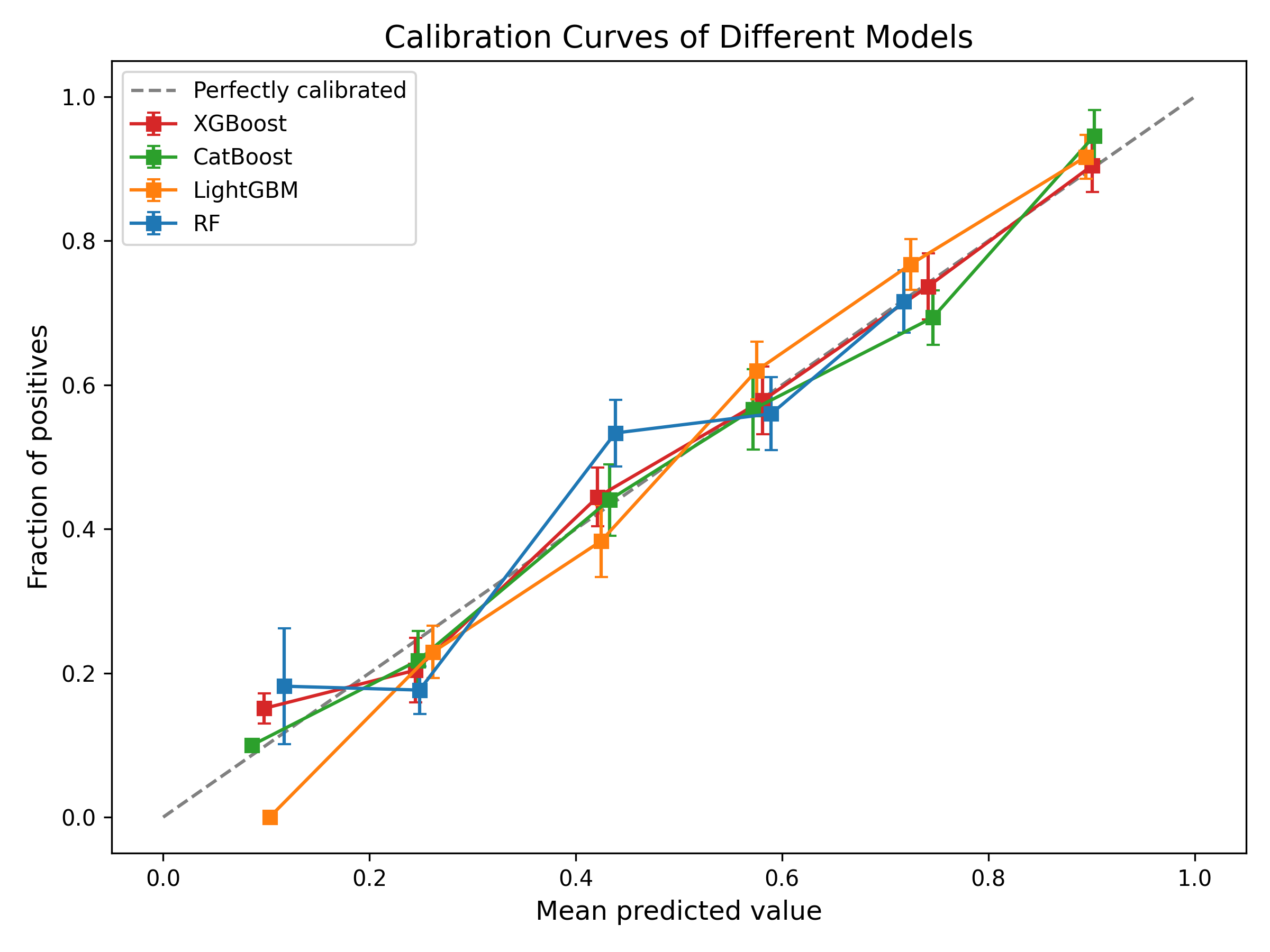

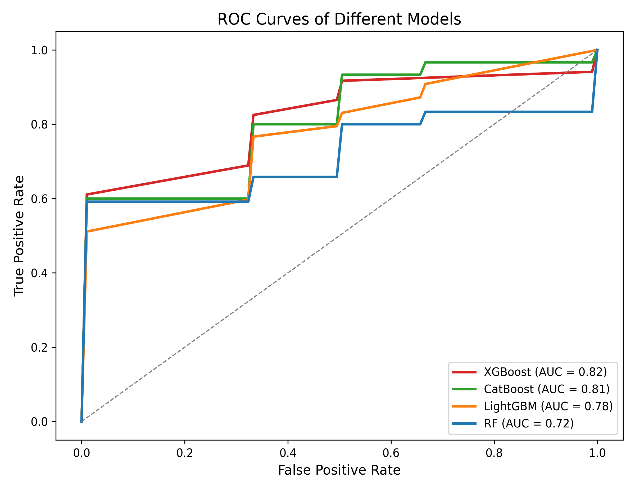

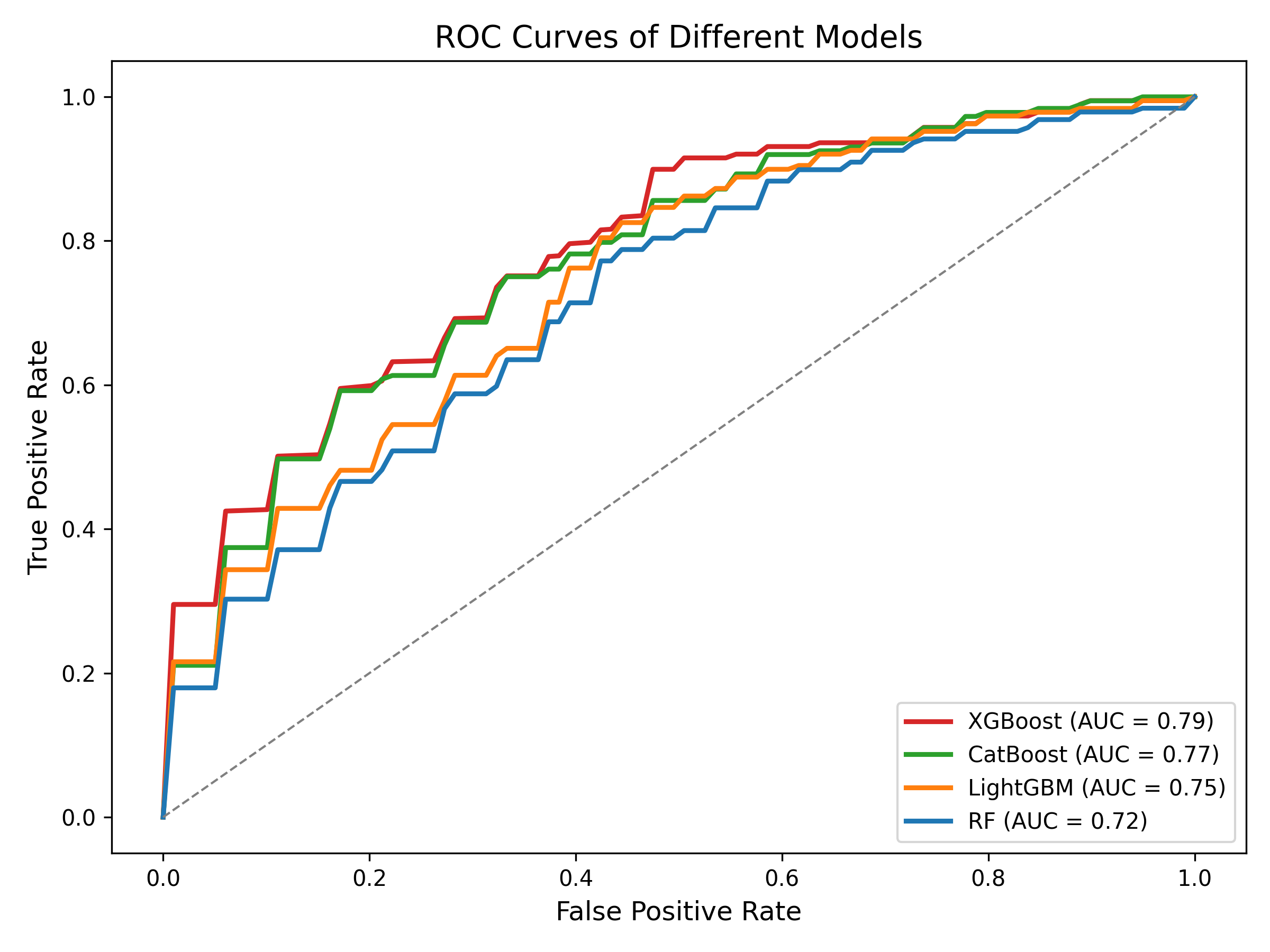

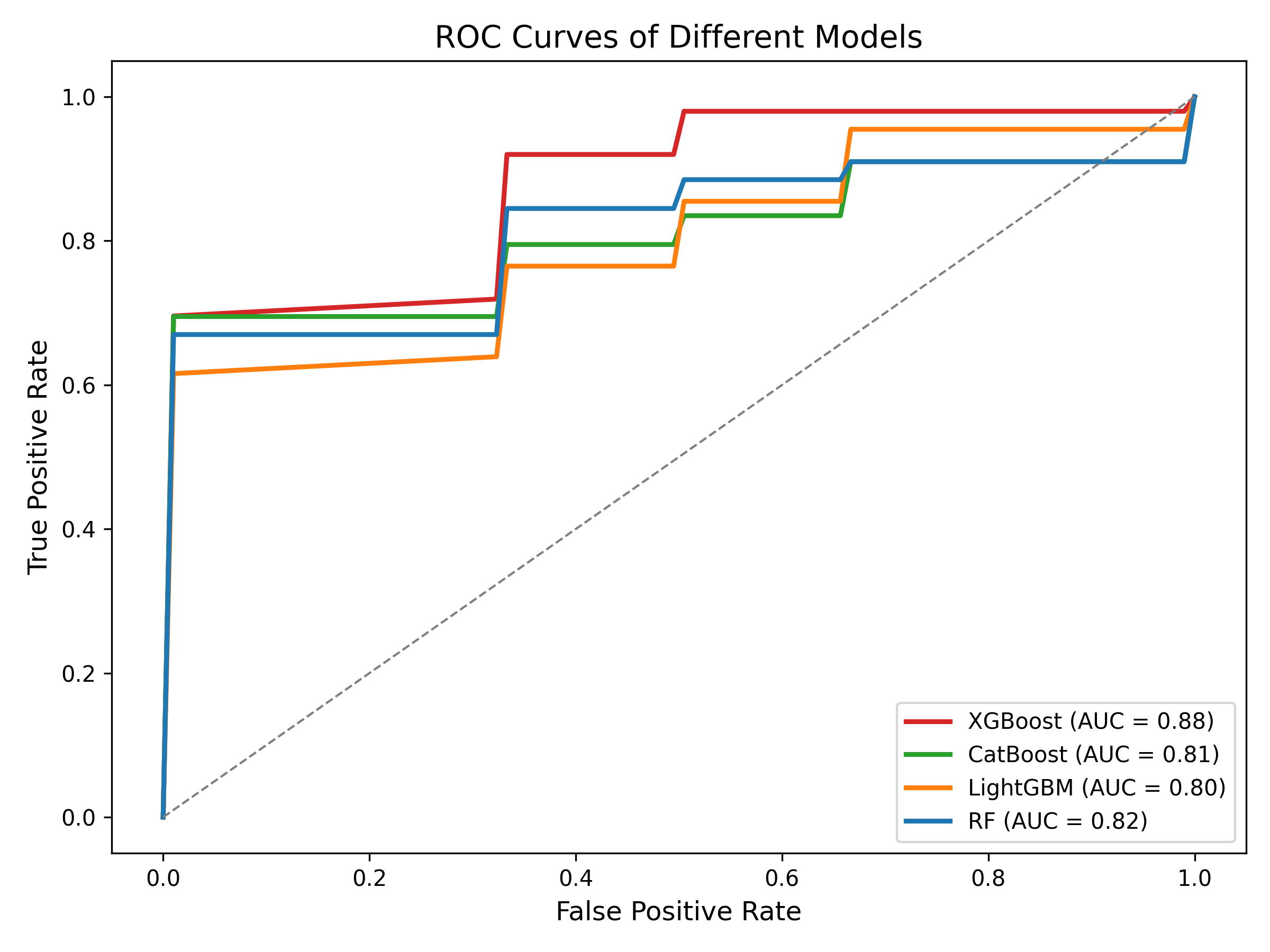

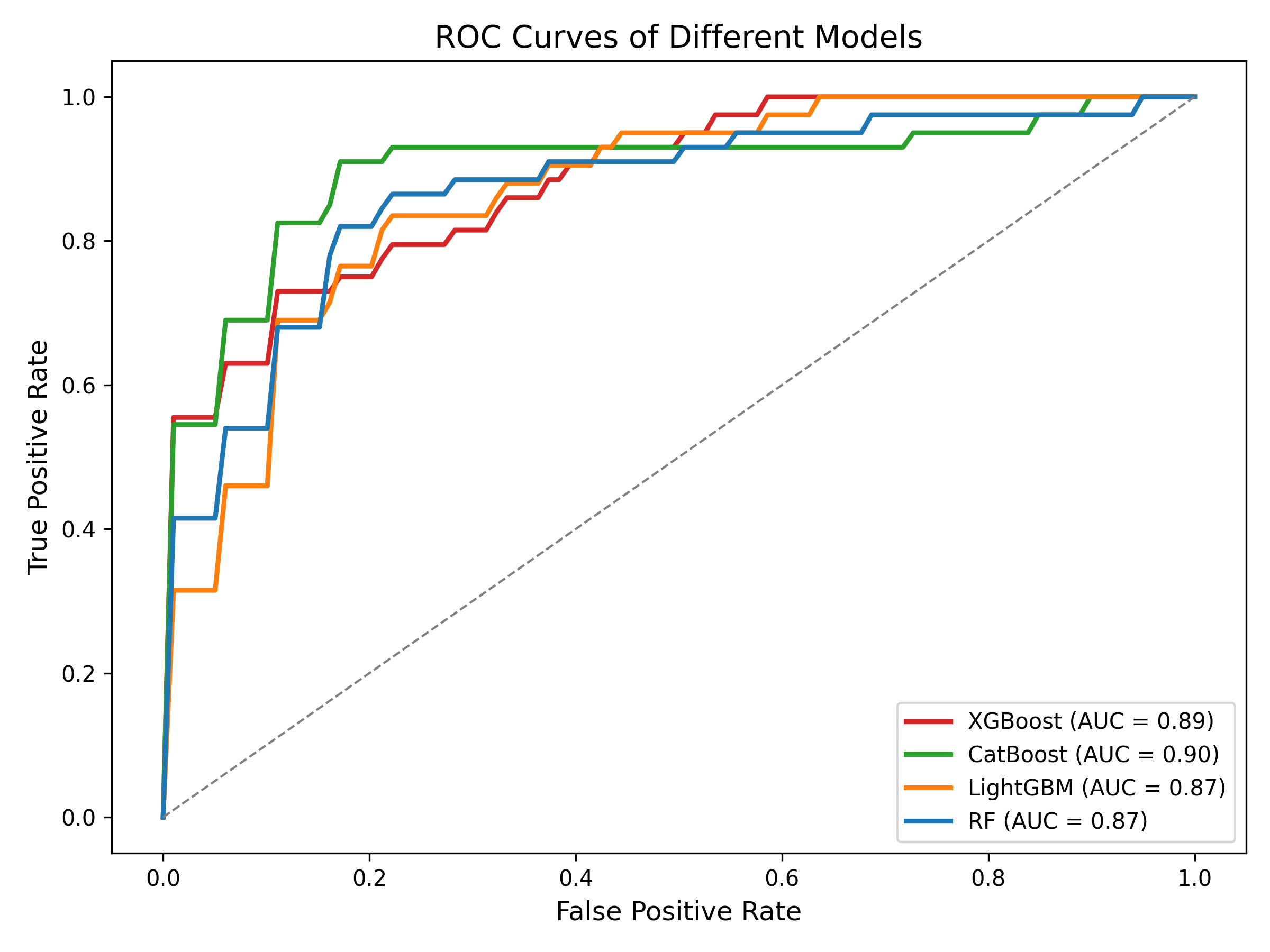

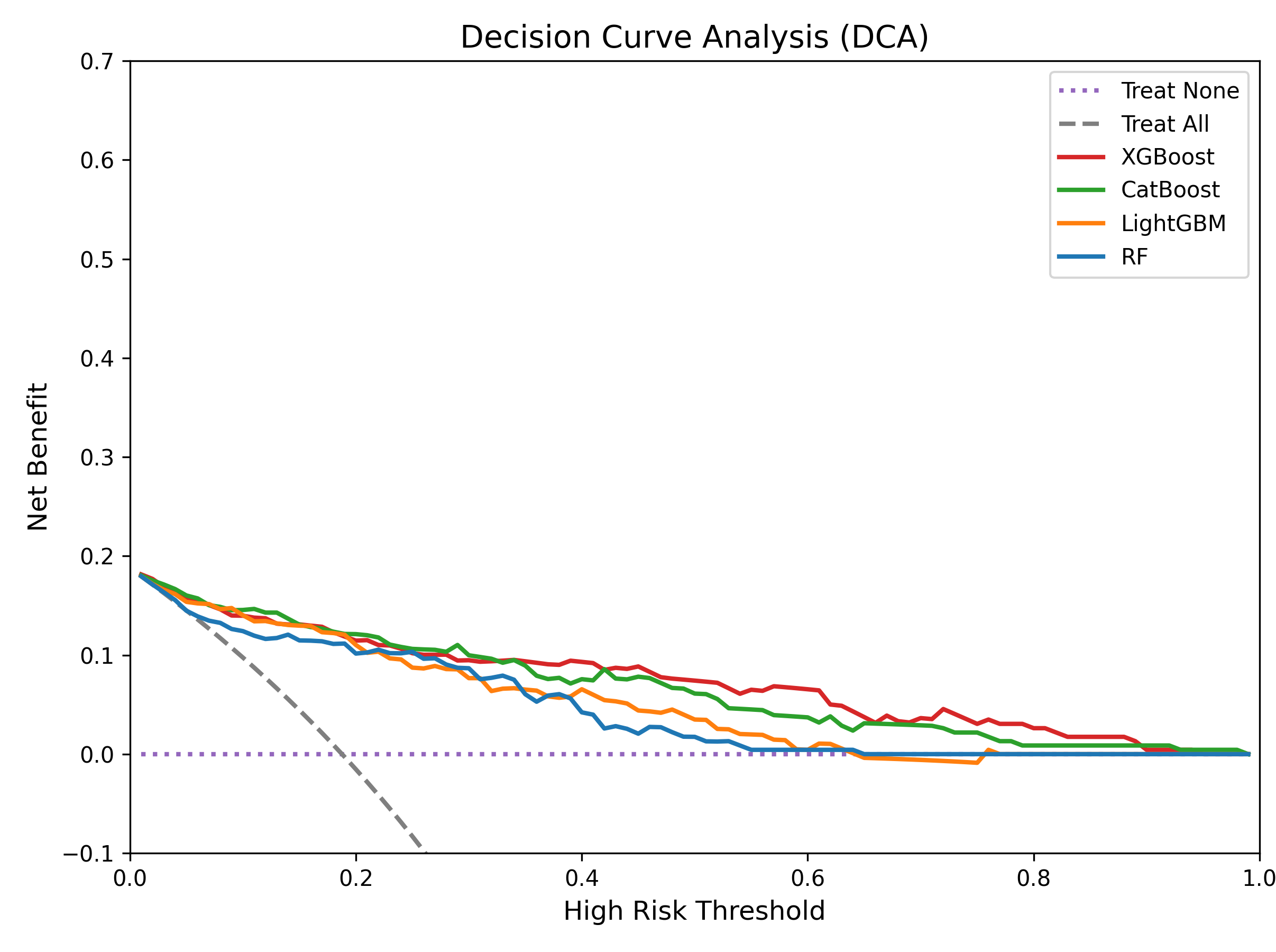

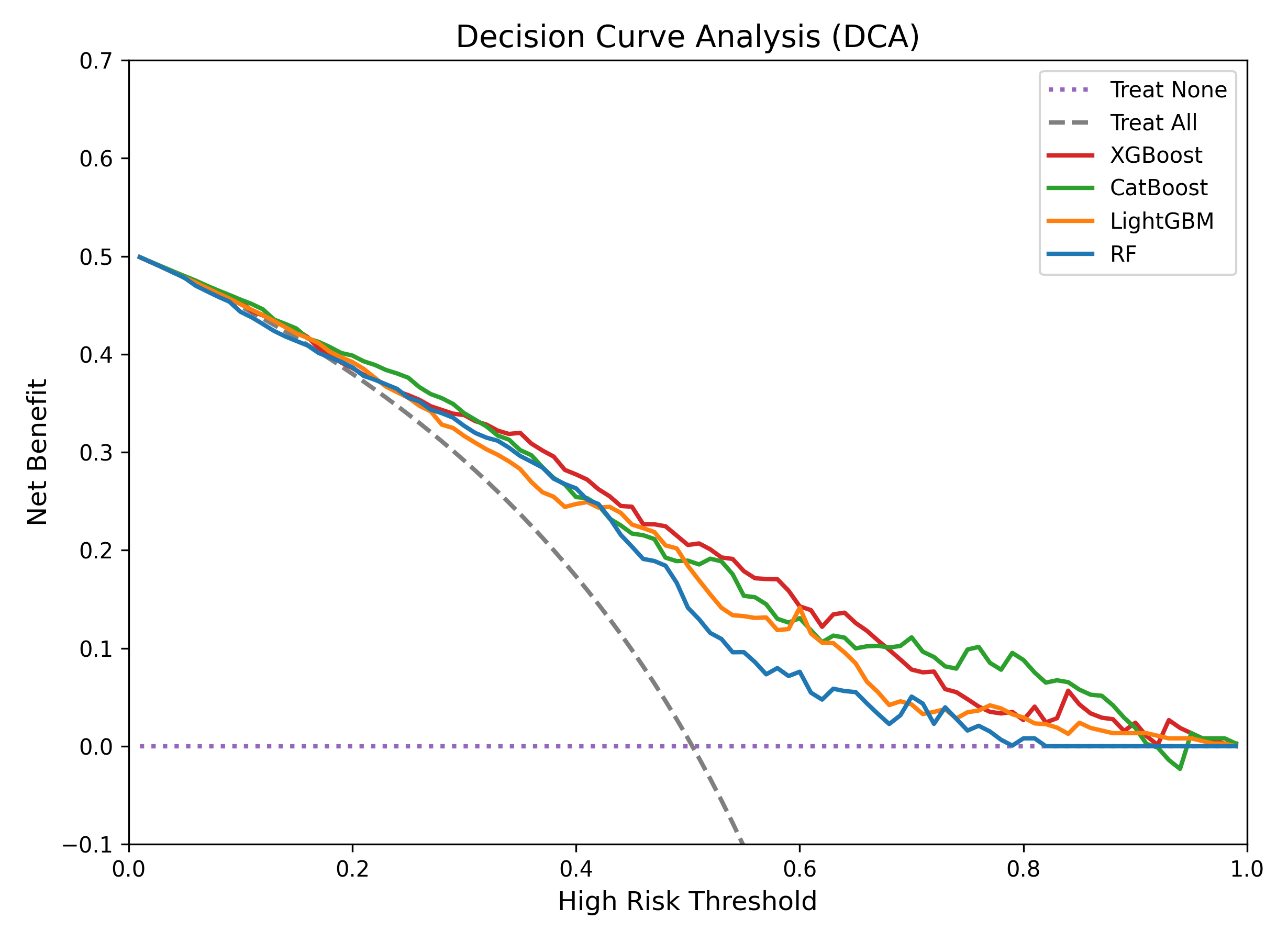


From left to right, the columns represent the four prediction tasks: pG, pN, rG, and rN. From top to bottom, the rows display the AUC, calibration plots, and decision DCA plots, respectively.

# Figure S4 Features selected by RFE


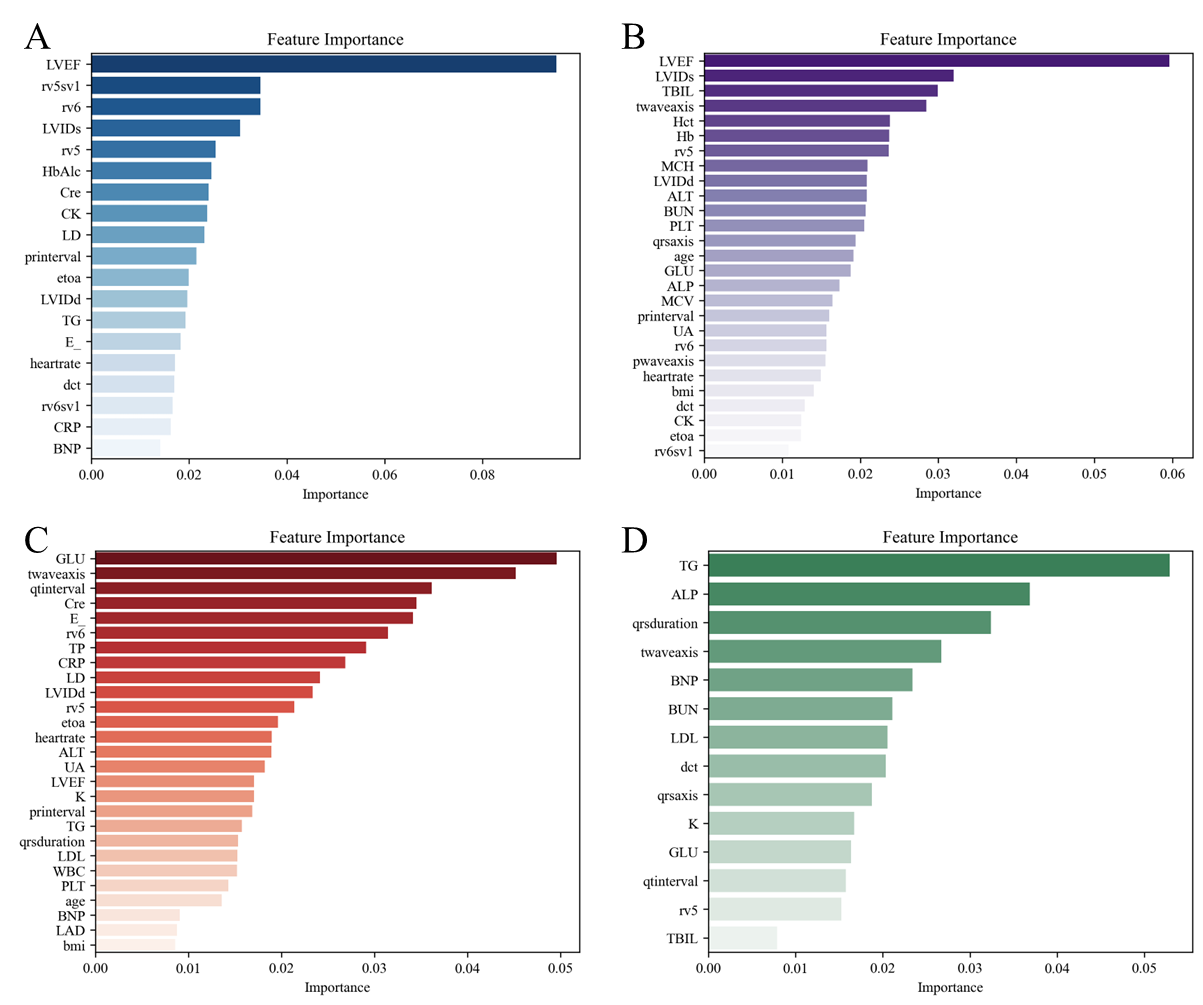


A. Features of pG^a^. B. Features of pN^b^. C. Features of rG^c^. D. Features of rN^d^.

^a^ pG: Preserved LVEF with good recovery task.

^b^ pN: Preserved LVEF with normal recovery task.

^c^ rG: Reduced LVEF with good recovery task.

^d^ rN: Reduced LVEF with normal recovery task.

# Figure S5 Features selected by LASSO


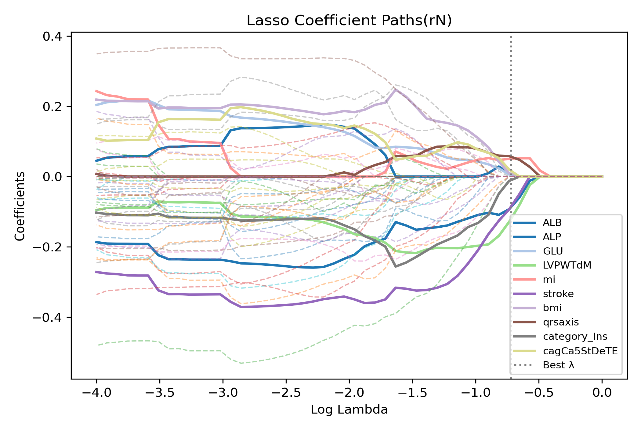

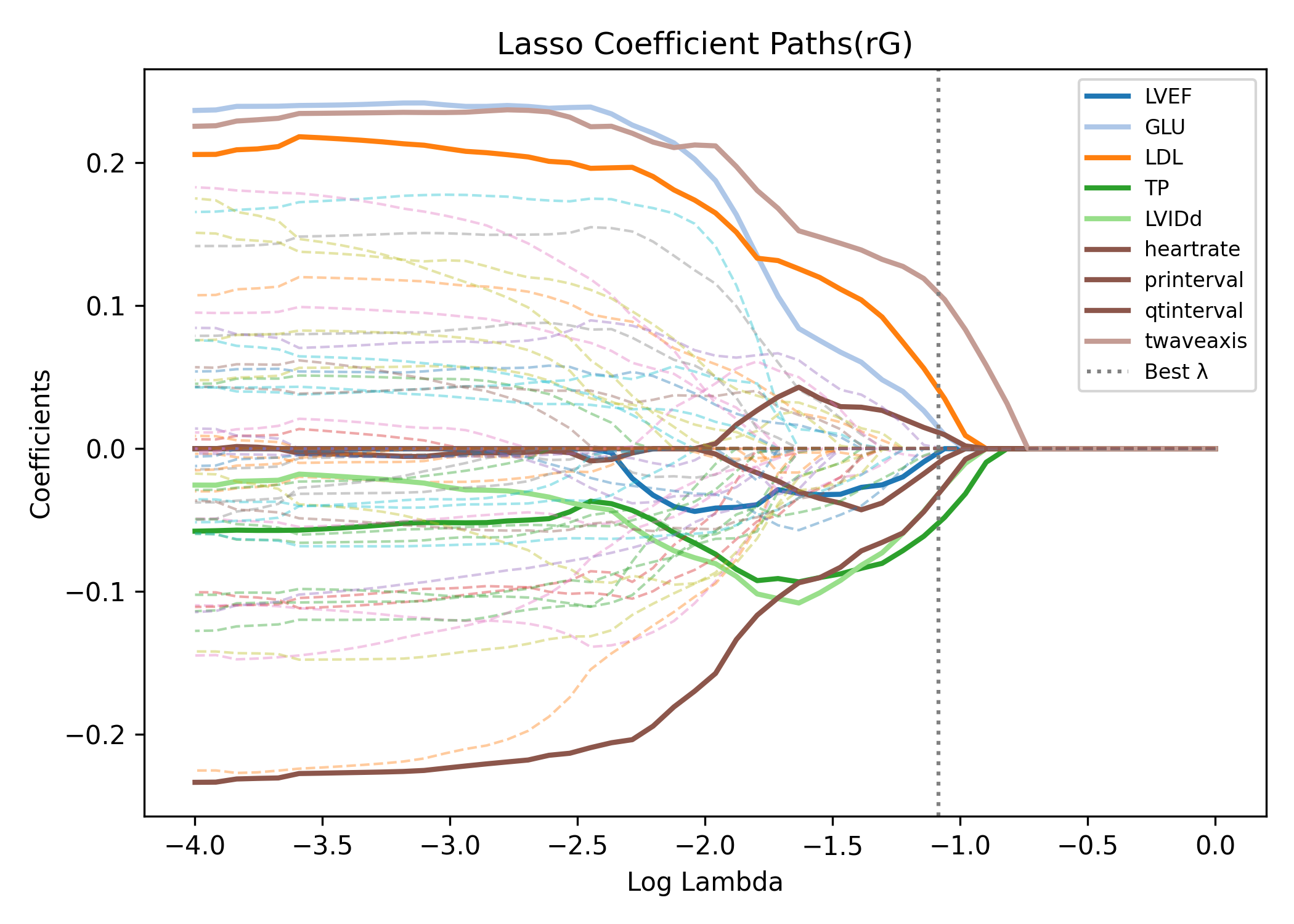

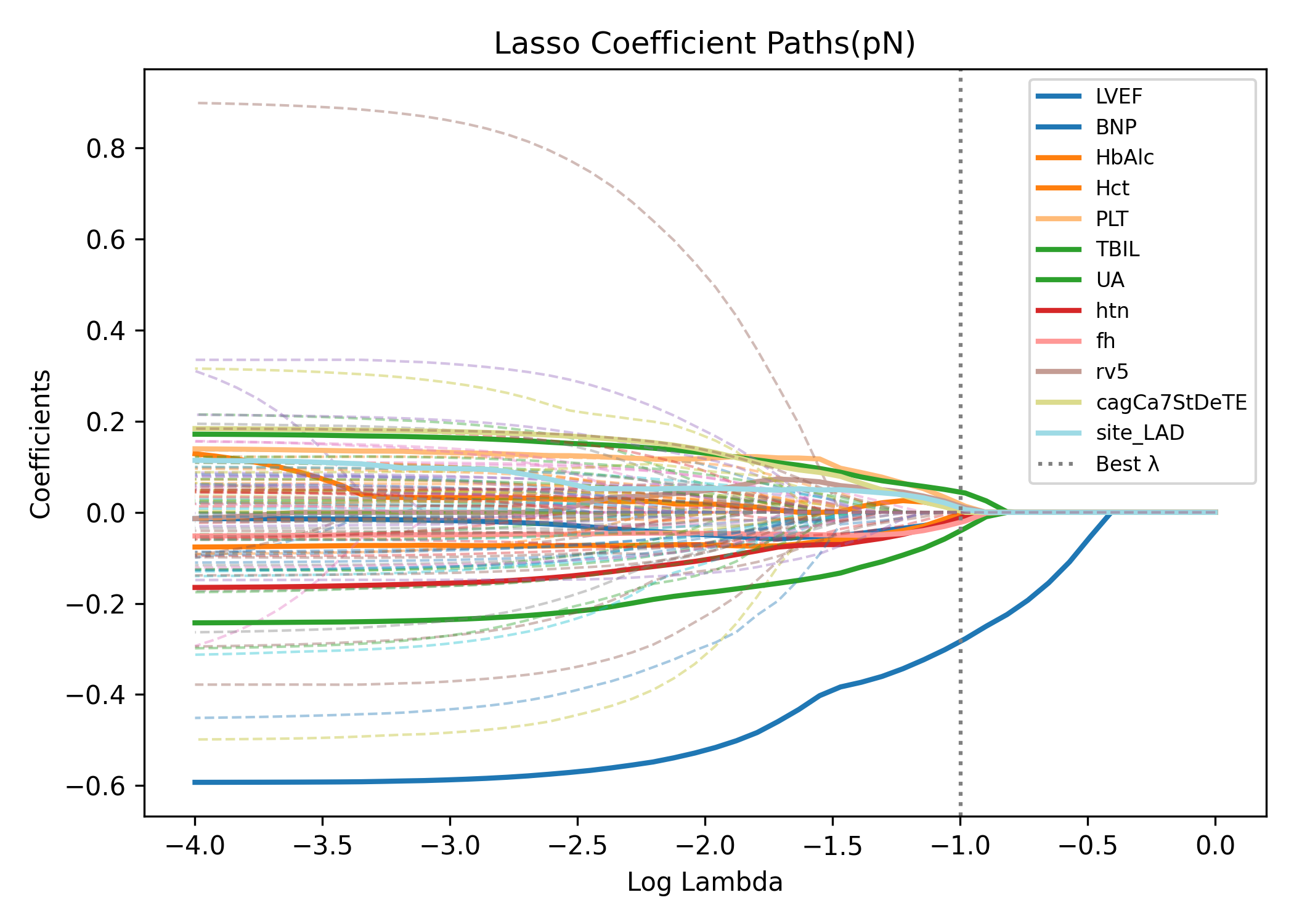

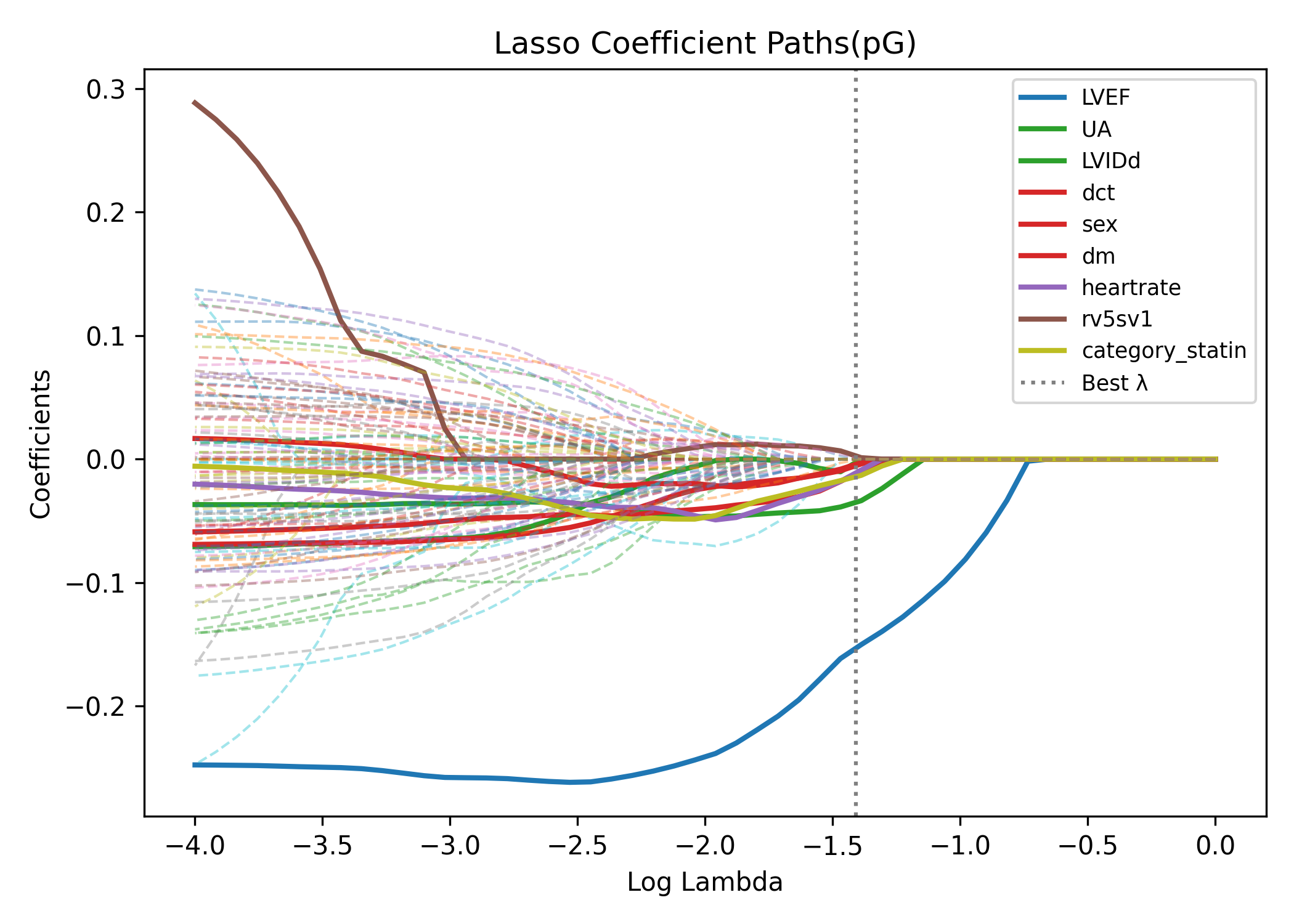


A

C

B

D

A. Features of pG^a^. B. Features of pN^b^. C. Features of rG^c^. D. Features of rN^d^.

^a^ pG: Preserved LVEF with good recovery task.

^b^ pN: Preserved LVEF with normal recovery task.

^c^ rG: Reduced LVEF with good recovery task.

^d^ rN: Reduced LVEF with normal recovery task.
